# Prevalence and clinical implications of coronary artery calcium scoring on non-gated thoracic computed tomography: a systematic review and meta-analysis

**DOI:** 10.1007/s00330-023-10439-z

**Published:** 2023-12-22

**Authors:** Maia Osborne-Grinter, Adnan Ali, Michelle C. Williams

**Affiliations:** 1 BHF Centre for Cardiovascular Science, University of Edinburgh, Edinburgh, UK; 2 University of Bristol, Bristol, UK; 3 School of Medicine, University of Dundee, Dundee, UK; 4 Edinburgh Imaging Facility QMRI, University of Edinburgh, Edinburgh, UK

**Keywords:** Coronary artery disease, X-ray computed tomography, Coronary artery calcifications

## Abstract

**Objectives:**

Coronary artery calcifications (CACs) indicate the presence of coronary artery disease. CAC can be found on thoracic computed tomography (CT) conducted for non-cardiac reasons. This systematic review and meta-analysis of non-gated thoracic CT aims to assess the clinical impact and prevalence of CAC.

**Methods:**

Online databases were searched for articles assessing prevalence, demographic characteristics, accuracy and prognosis of incidental CAC on non-gated thoracic CT. Meta-analysis was performed using a random effects model.

**Results:**

A total of 108 studies (113,406 patients) were included (38% female). Prevalence of CAC ranged from 2.7 to 100% (pooled prevalence 52%, 95% confidence interval [CI] 46–58%). Patients with CAC were older (pooled standardised mean difference 0.88, 95% CI 0.65–1.11, *p* < 0.001), and more likely to be male (pooled odds ratio [OR] 1.95, 95% CI 1.55–2.45, *p* < 0.001), with diabetes (pooled OR 2.63, 95% CI 1.95–3.54, *p* < 0.001), hypercholesterolaemia (pooled OR 2.28, 95% CI 1.33–3.93, *p* < 0.01) and hypertension (pooled OR 3.89, 95% CI 2.26–6.70, *p* < 0.001), but not higher body mass index or smoking. Non-gated CT assessment of CAC had excellent agreement with electrocardiogram-gated CT (pooled correlation coefficient 0.96, 95% CI 0.92–0.98, *p* < 0.001). In 51,582 patients, followed-up for 51.6 ± 27.4 months, patients with CAC had increased all cause mortality (pooled relative risk [RR] 2.13, 95% CI 1.57–2.90, *p* = 0.004) and major adverse cardiovascular events (pooled RR 2.91, 95% CI 2.26–3.93, *p* < 0.001). When CAC was present on CT, it was reported in between 18.6% and 93% of reports.

**Conclusion:**

CAC is a common, but underreported, finding on non-gated CT with important prognostic implications.

**Clinical relevance statement:**

Coronary artery calcium is an important prognostic indicator of cardiovascular disease. It can be assessed on non-gated thoracic CT and is a commonly underreported finding. This represents a significant population where there is a potential missed opportunity for lifestyle modification recommendations and preventative therapies. This study aims to highlight the importance of reporting incidental coronary artery calcium on non-gated thoracic CT.

**Key Points:**

*• Coronary artery calcification is a common finding on non-gated thoracic CT and can be reliably identified compared to gated-CT.*

*• Coronary artery calcification on thoracic CT is associated with an increased risk of all cause mortality and major adverse cardiovascsular events.*

*• Coronary artery calcification is frequently not reported on non-gated thoracic CT.*

**Supplementary Information:**

The online version contains supplementary material available at 10.1007/s00330-023-10439-z.

## Introduction

Cardiovascular disease is the most common cause of death around the world [1]. However, the majority of patient with coronary artery disease are asymptomatic and unaware of this diagnosis. The presence of coronary artery calcium (CAC) indicates that a patient has coronary artery disease, and this gives important information about prognosis beyond that provided by traditional cardiovascular risk factors [2]. An elevated coronary artery calcium score increases the risk of subsequent adverse cardiovascular events, even in asymptomatic patients [3]. However, CAC can also be assessed on thoracic computed tomography (CT) conducted for non-cardiac reasons.

Electrocardiogram (ECG) gating is used for dedicated CT to assess CAC in order to minimise coronary artery motion artefacts and optimise quantification [4]. However, CAC can also be identified and quantified on non-ECG gated CT performed for non-cardiac indications. Several previous studies have shown that CAC on non-gated thoracic CT is associated with subsequent cardiac events, including in populations undergoing lung cancer screening [5–8]. Recently published guidelines support the reporting of CAC on all CT of the chest [9, 10]. However, at present, incidental CAC is frequently not reported on CT scans performed for non-cardiac indications [11, 12].

This systematic review and meta-analysis aimed to assess the prevalence of incidental CAC identified on non-gated thoracic CT and its association with patient characteristics and adverse outcomes. Furthermore, we investigate the accuracy of non-gated thoracic CT compared to gated cardiac CT for the assessment of CAC and the frequency of reporting of incidental CAC on non-gated CT.

## Methods

### Information sources and search terms

PRISMA reporting guidelines were followed for this meta-analysis (Supplementary Table 1) and the protocol was registered (PROSPERO CRD42022342234). PubMed, Medline and Embase were searched to July 2022 using the terms computed tomography, non-gated, coronary and calcification, and synonyms. Full search terms are listed in the supplementary information (Supplementary Table 2). Reference lists from relevant review articles and all eligible studies were also reviewed for relevant articles.

### Study selection

Two reviewers (M.O.G, A.A) participated in literature selection. Studies were included if they analysed one of the following topics about coronary artery calcium scoring (CACS) on non-gated thoracic CT: (1) prevalence of CAC on non-gated thoracic CT, (2) agreement between non-gated and gated CT, (3) comparison of patient characteristics with or without CAC on non-gated thoracic CT, (4) prognostic performance of CAC to predict adverse events on non-gated thoracic CT, (5) reporting practices of CAC on non-gated thoracic CT.

Conference proceedings, case reports, editorials, letters, opinion pieces and studies without English versions were excluded. Titles and abstracts were screened and full texts were obtained. Two investigators independently assessed articles for eligibility and quality, with disagreement settled by consensus. When multiple publications based on the same trial were identified, only the largest sample size was included to avoid duplicate reporting.

### Data extraction

A standardised data extraction form was used to collect study and participant characteristics, methodology and study results. For all studies, information was collected concerning study design, CT technology, and participant demographic characteristics. The prevalence and severity of CAC was recorded, using continuous or categorical metrics as available. When available, the proportion of subjects with a CAC of 0 versus above 0, and below and above 400 Agatston units (AU) were extracted. A ‘high’ CACS was defined as Agatston score > 300 AU or a visual ordinal score of severe. Information on reporting practices and agreement between gated and non-gated CT were recorded. For studies assessing agreement, correlation coefficients were extracted for continuous data and weighted-Cohen *K* was calculated for categorical data.

To calculate the prognostic significance of CAC on thoracic CT, information on cardiovascular events during follow-up was extracted. The number of events were recorded for all-cause mortality, coronary heart disease death and major adverse cardiovascular events (MACEs) including cardiovascular death, non-fatal myocardial infarction, stroke, new-onset arrhythmia and heart failure, where possible, raw data, adjusted and unadjusted hazard ratios with 95% confidence intervals (CI) were recorded.

### Study quality assessment

Two reviewers evaluated study quality. Studies assessing agreement between non-gated and gated CT were evaluated using the Quality Assessment of Diagnostic Accuracy Studies 2 [13] method with each domain scored from 1 (fulfilled) to 0 (unmet) and a total possible score of 7. For studies evaluating the presence of CAC, reporting practices and associations with demographic characteristics, study quality was assessed using the National Heart, Lung and Blood Institute (NIH) Quality Assessment Tool for Observational and Cross-sectional Studies [14]. Each domain was scored from 1 (fulfilled) to 0 (unmet) with a total possible score of 14. Seventeen studies of prognosis were evaluated with the American College of Cardiology Foundation/American Heart Association (ACCF/AHA) 8 criteria tool [14] (12). It gives a total score of 16, with ≥ 11 considered high quality, 7–10 moderate quality and ≤ 6 low quality. Five studies of prognosis were assessed using the NIH Quality Assessment Tool for Case-Cohort studies, with domains scored as 1 (fulfilled) to 0 (unmet) and a total possible score of 12 [14].

### Data synthesis and statistical analysis

Statistical analysis was performed using R version 1.3.959 (R Foundation). A 2-sided *p* value < 0.05 was considered significant. Where age was reported as median with interquartile range, assessment for skewness was performed using the method reported by Shi et al [15]. If the skew of the data was significant, it was excluded from the analysis and if it was not, mean and standard deviation was calculated using the method reported by Shi et al [16] and Luo et al [17]. Meta-analysis was performed using the metafor package [18]. Pooling calculations for agreement between calcium assessment methods was performed using the Hedges-Vevea random effects model. *Q* statistics and *I*^2^ index were used to test for heterogeneity with a 2-sided *p* value < 0.1 or > 50% indicating heterogeneity, respectively. For categorical variables, proportion and 95% CI are presented. The relative risk (RR) and 95% CI was calculated for the risk of events in patients with and without CAC. Odds ratios (ORs) and 95% CI were calculated for the presence of cardiovascular risk factors in patients with and without CAC. Standardised mean difference was calculated for continuous variables. Publication bias was assessed using the Begg and Mazumdar rank correlation and Egger regression test if the effect size was 3 or more in the included studies.

## Results

### Study selection

**Fig. 1:**
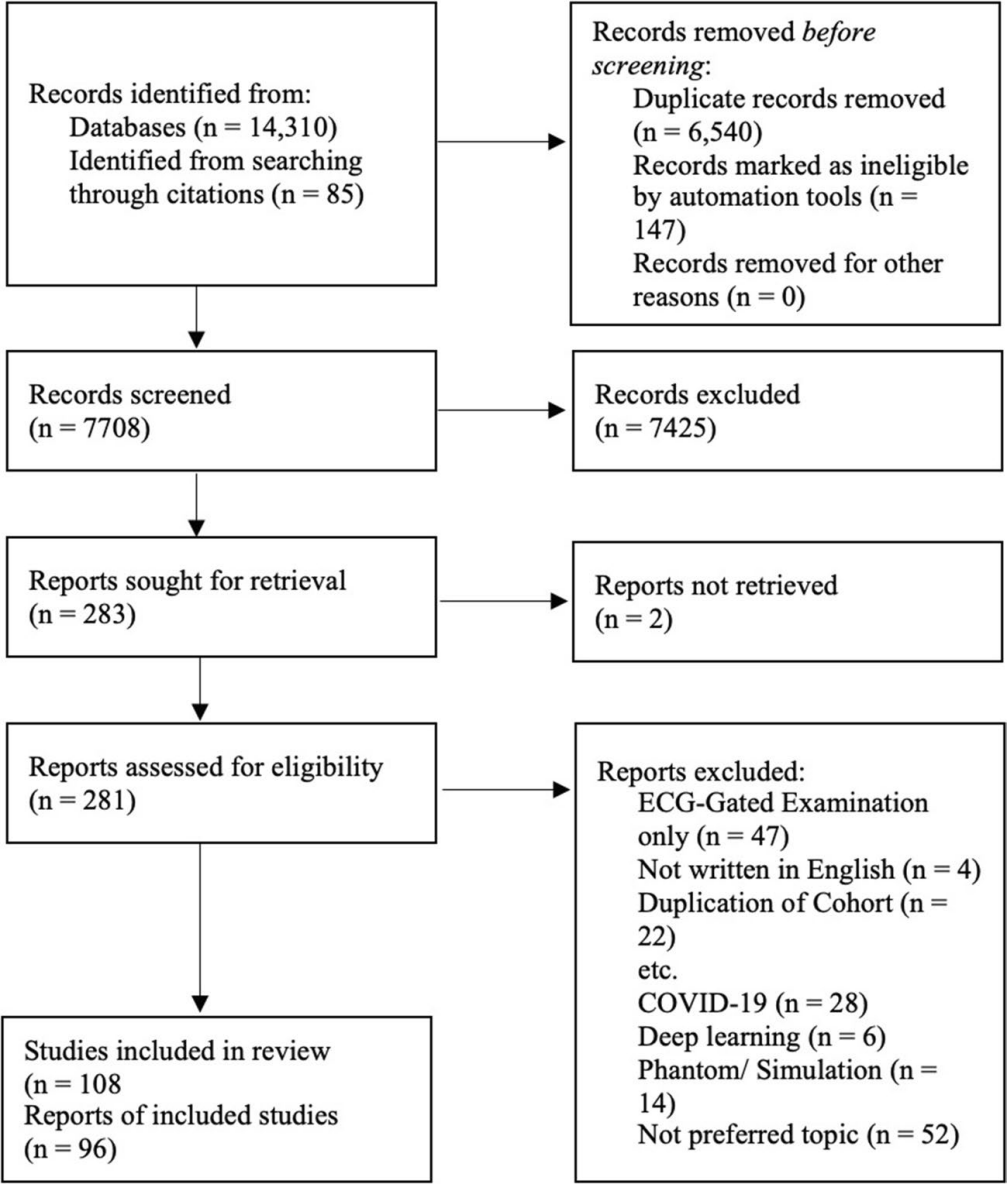
PRISMA algorithm of papers included for systematic review and meta-analysis

After removal of duplicates, 7708 papers were identified (Fig. 1). Of these, 108 studies were included in the systematic review [11, 19–125] and meta-analysis was performed on 96 studies assessing CAC prevalence, 23 studies comparing non-gated to gated CT, 33 studies providing information on participant characteristics, 41 studies assessing the prognostic implication of CAC and 12 studies assessing reporting practices (Supplementary Table 4).

### Study characteristics

The systematic review included 114,036 participants (range of mean age 46.4 to 74 years), comprising 63,115 men (55%), 43,138 females (38%) and 7743 (7%) individuals without indicated sex (Table 1). Nineteen studies were prospective and 92 studies were retrospective. A variety of CT modalities were used ranging from single slice to 256-row multidetector CT, and including attenuation correction CT for positron emission tomography. Six studies used electron beam CT. Low-dose CT acquisition was used in 44 studies and normal dose CT in 68 studies. CAC was assessed with the Agatston method [126] in 54 studies, with a visual ordinal or Weston scale in 30 studies, or with binary assessment of its presence or absence in 26 studies.

**Table 1:** Table of characteristics of studies included for systematic review and meta-analysis

Analysis type		Total population	Population cohort	Men (%)	Age (mean) ± SD	BMI (mean) ± SD	Hypertension (%)	Hypercholesterolaemia (%)	Diabetes (%)	Smoking history (%)
All patients		114,036	1 × bronchiectasis cohort, 5 × cardiac disease cohorts, 5 × COPD cohorts, 1 × COVID cohort, 6 × CTPA cohorts, 41 × general cohorts, 3 × HIV/AIDS cohorts, 30 × lung cancer screening cohorts, 12 × oncology cohorts, 4 × transplant cohorts	55.4	60.7 ± 6.4	25.7 ± 1.8	39.6	33.5	16.3	58.1
Prevalence of CACS		89,006	1 × bronchiectasis cohort, 2 × cardiac disease cohorts, 4 × COPD cohorts, 1 × COVID cohort, 6 × CTPA cohorts, 35 × general cohorts, 3 × HIV/ AIDS cohorts, 28 × lung cancer screening cohorts, 12 × oncology cohorts, 4 × transplant cohorts	56.2	60.3 ± 6.4	26.1 ± 1.5	39.9	34.1	13.2	65.9
Assessment of demographic characteristics	All	54,088	1 × bronchiectasis cohorts, 2 × CTPA cohorts, 16 × general cohorts, 2 × HIV/ AIDs cohorts, 9 × lung cancer screening cohorts, 2 × oncology cohorts, 1 × transplant cohort	52.9	60.5 ± 9.1	26.5 ± 1.4	44.5	39.5	13.3	76.2
	Sex	54,088	1 × bronchiectasis cohorts, 2 × CTPA cohorts, 16 × general cohorts, 2 × HIV/ AIDs cohorts, 8 × lung cancer screening cohorts, 2 × oncology cohorts, 1 × transplant cohort	52.9	60.5 ± 9.1	26.5 ± 1.4	44.5	39.5	13.3	76.2
	Age	16,972	1 × bronchiectasis cohort, 1 × CTPA cohort, 7 × general cohorts, 1 × HIV/AIDs cohort, 3 × lung cancer screening cohorts, 1 × oncology cohort	53.5	59.3 ± 10.2	26.9 ± 1.2	46.4	38.6	16.4	52.7
	Body mass index	7328	1 × HIV/AIDS cohort, 3 × general cohorts, 1 × lung cancer screening cohort	57.9	57.3 ± 10.8	26.6 ± 0.7	35.3	54.8	11.5	94.8
	Hypertension	21,446	1 × bronchiectasis cohort, 2 × CTPA cohorts, 10 × general cohorts, 2 × HIV/AIDS cohorts, 5 × lung cancer screening cohorts, 1 × oncology cohort, 1 × transplant cohort	53.8	61.3 ± 7.6	27.2 ± 0.9	47.1	38.1	16.4	56.1
	Hypercholesterolaemia	15,585	1 × bronchiectasis cohort, 2 × CTPA cohorts, 10 × general cohorts, 1 × HIV/AIDS cohort, 5 × lung cancer screening cohorts, 1 × oncology cohort, 1 × transplant cohort	51.1	62.7 ± 6.7	27.6 ± 0.6	51.5	38.1	18.3	38.9
	Diabetes	25,696	1 × bronchiectasis cohort, 2 × CTPA cohorts, 10 × general cohorts, 2 × HIV/AIDS cohorts, 7 × lung cancer screening cohorts, 1 × oncology cohort, 1 × transplant cohort	52.8	61.3 ± 7.6	27.2 ± 0.9	47.1	38.1	13.9	63.1
	Smoking history	29,312	1 × bronchiectasis cohort, 2 × CTPA cohorts, 9 × general cohorts, 2 × HIV/AIDs cohorts, 6 × lung cancer screening cohorts, 1 × oncology cohort, 1 × transplant cohort	53.0	60.0 ± 10.0	26.9 ± 1.2	46.1	35.6	16.0	69.2
Prognostic implications	All	51,582	1 × bronchiectasis cohort, 2 × COPD cohorts, 1 × COVID cohort, 4 × CTPA cohorts, 15 × general cohorts, 8 × lung cancer screening cohorts, 7 × oncology cohorts, 2 × transplant cohorts	64.5	60.8 ± 4.9	29.0 ± 5.0	36.8	36.1	13.8	66.2
	CACS high vs low	22,771	1 × COPD cohort, 1 × CTPA cohort, 4 × general cohorts, 1 × lung cancer screening cohort, 2 × oncology cohorts, 1 × transplant cohort	70.2	60.4 ± 5.6	26.0 ± 3.9	34.6	39.3	11.9	60.2
	Lung cancer screening cohorts	27,455	5 × lung cancer screening	43.8	60.2 ± 2.6	26.0 ± 3.9	29.4	41.4	8.8	49.6
Reporting of CACS		5350	1 × CTPA cohort, 7 × general cohorts, 1 × lung cancer screening cohort, 2 × oncology cohorts	67.0	63.4 ± 5.6	27.1 ± 6.1	50.7	41.4	20.8	75.8
Agreement of gated and non-gated CT	All	6537	3 × cardiac disease cohorts, 1 × COPD cohort, 11 × general cohorts, 8 × oncology cohorts	54.6	60.5 ± 5.7	25.4 ± 1.3	40.7	31.7	22.0	52.2
	Correlation	5181	1 × cardiac disease cohort, 1 × COPD cohort, 6 × general cohorts, 7 × oncology cohorts	58.7	59.6 ± 6.7	26.1 ± 1.0	38.6	32.7	22.1	34.7
	Categories	5396	2 × cardiac disease cohorts, 1 × COPD cohort, 8 × general cohorts, 6 × oncology cohorts	52.4	60.0 ± 5.1	25.1 ± 1.1	40.2	35.4	26.4	52.0

*CACS* coronary artery calcium score, *COPD* chronic obstructive pulmonary disease, *CTPA* computed tomography pulmonary angiography

### Prevalence of coronary artery calcification on non-gated CT

**Fig. 2:**
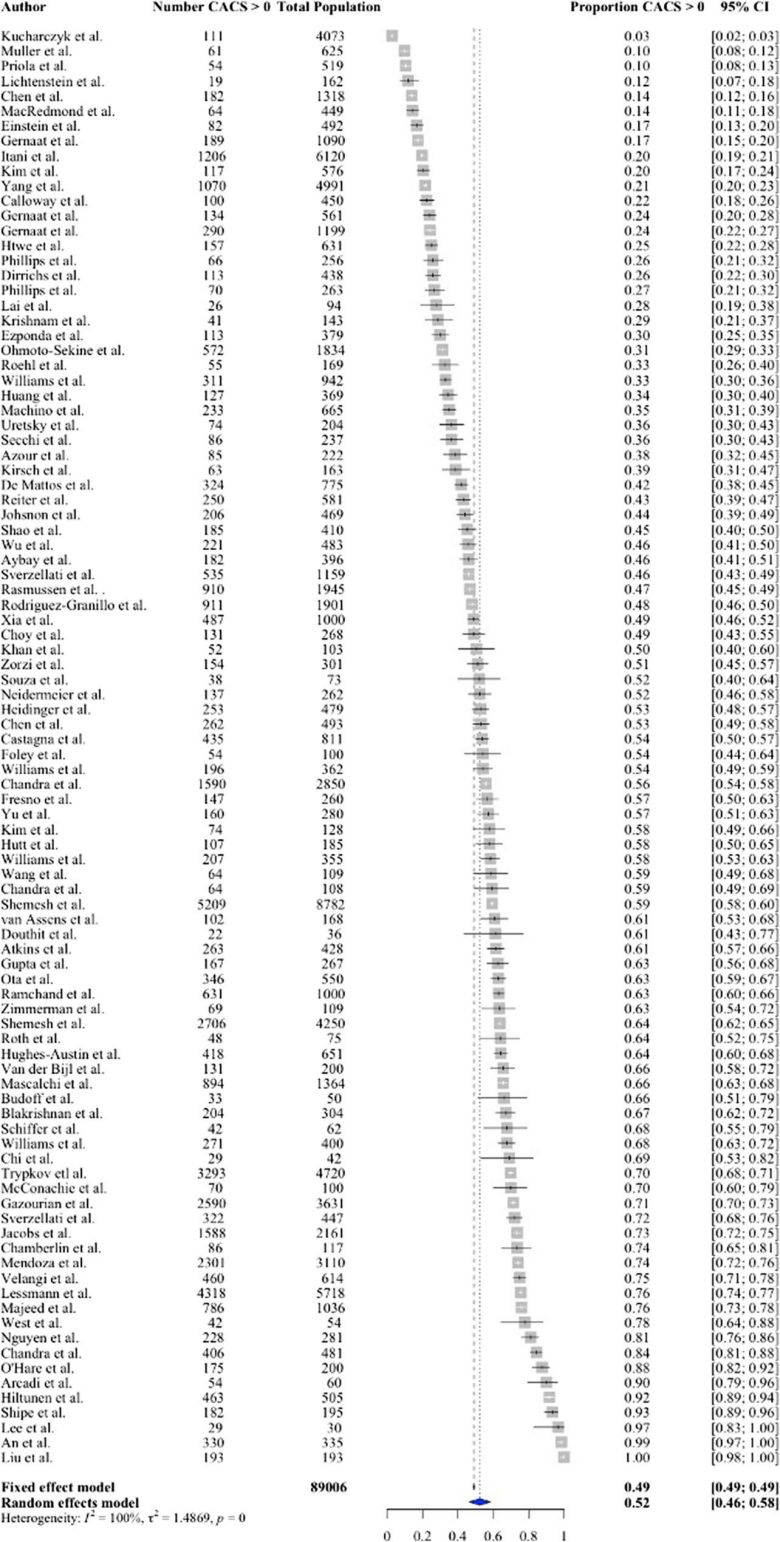
Forest plot of the prevalence of incidental calcium on non-gated thoracic CT

Prevalence of incidental CAC on non-cardiac CT was reported in 94 studies, including 89,006 patients. The prevalence ranged from 2.7 to 100% (Fig. 2) with a pooled prevalence of 52% (95% CI 46 to 58%).

There was moderate heterogeneity between the included studies (*I*^2^ > 50%, *p* = 0) with higher CAC prevalence in studies of older patients and those with a higher prevalence of men. No publication bias was found in the pooling (Eggers: *p* = 0.23, Begg’s:* p* = 0.31). All 66 studies that evaluated the incidence of CAC, reporting practices for CAC or cardiovascular risk factors, were of high or moderate quality with sub-optimal scores present for 9 domains (Supplementary Table 3).

### Impact of demographic characteristics on coronary artery calcification

Information on cardiovascular risk factors was available in 33 studies (sex in 32 studies, age in 14 studies, body mass index (BMI) in 5 studies, diabetes mellitus in 24 studies, hypercholesterolaemia in 21 studies, hypertension in 22 studies and smoking history in 22 studies). All of these studies showed heterogeneity in the pooling calculation (*Q* test *p* < 0.001 and *I*^2^ > 50%). No publication bias was found in the pooling (Eggers: *p* = 0.50, *p* = 0.25, *p* = 0.12, *p* = 0.15, *p* = 0.11, *p* = 0.67, *p* = 0.83).

Mean age was 60.3 ± 10.3 years and patients with CACS > 0 were older than those without CAC (pooled standardised mean difference 0.88, 95% CI 0.65 to 1.11, *p* < 0.001, Supplementary Fig. 1). Of the 106,293 patients where sex was reported, 59.4% were male and 40.6% were female. Men were more likely to have CAC than women (pooled odds ratio 1.95, 95% CI 1.55 to 2.45, *p* < 0.001, Supplementary Fig. 2). The presence of diabetes mellitus (pooled odds ratio 2.63, 95% CI 1.95 to 3.54, *p* < 0.001, Supplementary Fig. 3), hypercholesterolaemia (pooled odds ratio 2.28, 95% CI 1.33 to 3.93, *p* < 0.01, Supplementary Fig. 4), and hypertension (pooled odds ratio 3.89, 95% CI 2.26 to 6.70, *p* < 0.001, Supplementary Fig. 5) was all associated with an increased likelihood of having CAC. However, there was no difference in smoking history (pooled odds ratio 1.35, 95% CI 0.98 to 1.85, *p* = 0.06, Supplementary Fig. 6) or BMI between those with and without CAC (pooled standardised mean difference 0.05, 95% CI − 0.34 to 0.44, *p* = 0.74, Supplementary Fig. 7).

### Prognostic implications of CAC on non-gated CT

Forty studies (51,582 patients) reported cardiovascular events or all-cause mortality in patients with and with out CAC. Patients were followed up for a mean of 51.6 ± 27.4 months.

**Fig. 3:**
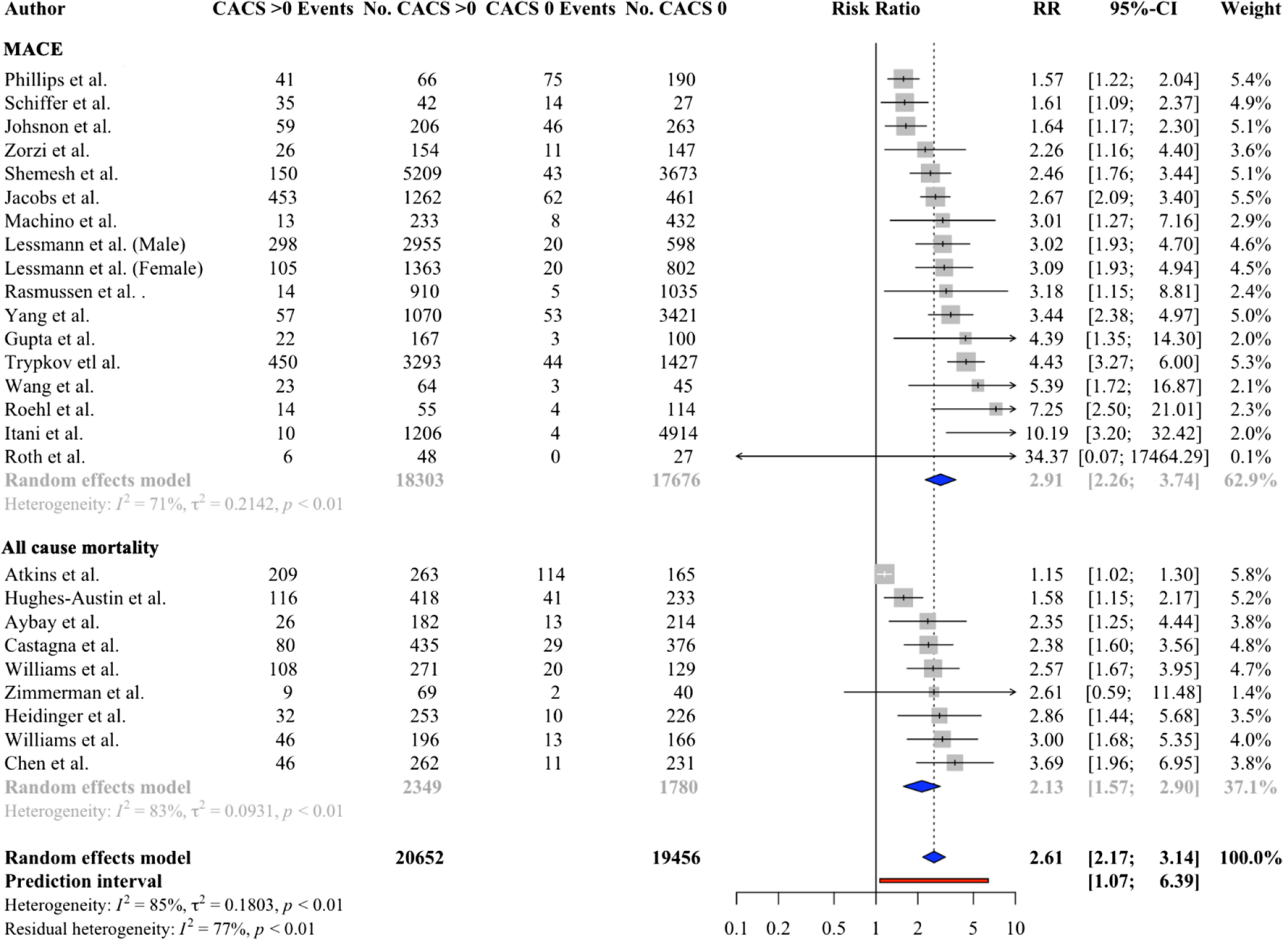
Forest plot showing the relative risk of major adverse cardiovascular events (MACEs), all-cause mortality, and all events for patients with CACS 0 and CACS > 0

Compared the patients without CAC, patients with CAC had an increase risk of all-cause mortality (pooled relative risk 2.13, 95% CI 1.57 to 2.90, *p* = 0.004), MACE (pooled relative risk 2.91, 95% CI 2.26 to 3.93, *p* < 0.001) and combined all-cause mortality and MACE (pooled relative risk 2.61, 95% CI 2.17 to 3.74, *p* < 0.001, Fig. 3). There was heterogeneity amongst studies reporting all cause mortality or MACE (*I*^2^ > 50%, *Q* test *p* < 0.001). Publication bias could not be assessed for all-cause mortality due to insufficient sample size. No publication bias was found in the pooling calculations for MACE or combined all-cause mortality and MACE (Eggers test *p* = 0.08 and *p* = 0.17, respectively).

There were 10 studies which reported cardiovascular events or all-cause mortality in patients with different categories of CAC severity. There was an increased risk of all cause mortality and MACE in patients with a ‘high’ CACS compared to those with a ‘low’ CACS (RR 3.92, 95% CI 2.50 to 6.14, *p* < 0.001, Supplementary Fig. 8). There was heterogeneity in the pooling calculation (*I*^2^ > 50%, *Q* test *p* < 0.001) but in the pooling calculation no publication bias was found (*p* = 0.92).

The clinical implications of CAC in patients undergoing lung cancer screening were reported in 5 studies. Compared to patients without CAC, patients with CAC who were undergoing lung cancer screening had an increased risk of combined MACE and all cause mortality (pooled relative risk 3.27, 95% CI 1.88 to 5.68, *p* = 0.004). There was no heterogeneity amongst these studies (*I*^2^ < 50%, *Q* test *p* = 0.23 and) but publication bias could not be assessed due to insufficient sample size.

All prognostic studies were of high or moderate quality, with suboptimal scores present due to limited sample sizes (Supplementary Fig. 3).

### Reporting of incidental CAC on non-gated CT

**Fig. 4:**
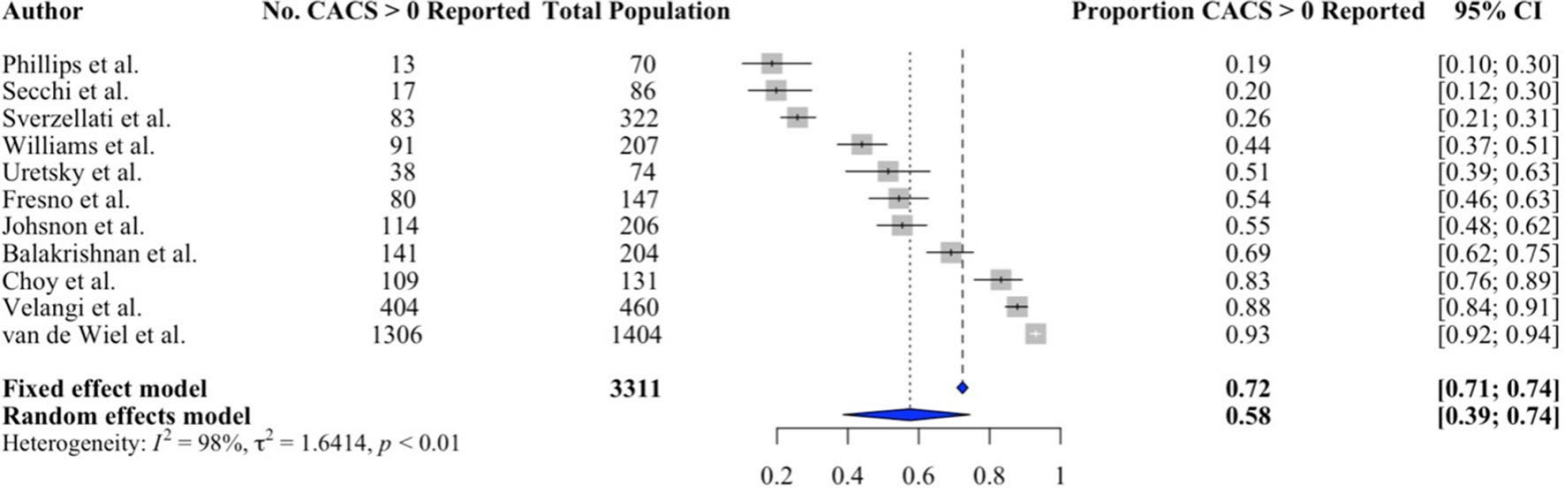
Forest plot of the proportion of reports that include incidental coronary artery calcium when it is present

Eleven studies (5350 patients) assessed whether incidental CAC on non-gated CT was reported. When CAC was present, it was reported in between 18.6% and 93% of CT reports. The pooled proportion of reports that included mention of CAC when present was 57% (95% CI 0.39 to 0.74,* p* < 0.01, Fig. 4). These studies had significant heterogeneity (*I*^2^ = 98%, *p* < 0.001) but no publication bias was found in the pooling (Eggers: *p* = 0.17; Begg: *p* = 0.70).

### Agreement between gated and non-gated CT

**Fig. 5:**
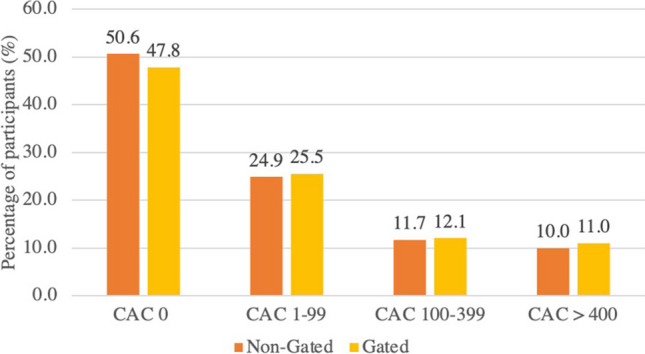
Bar chart showing the percentage of participants with CAC = 0, CAC 1–99, CAC 100–399 and CAC > 400 reported on non-gated and ECG-gated CT

Twenty-three studies (6537 patients) provided information on the diagnostic accuracy of non-gated CT compared to gated CT, of which 47.8% had no CAC, 25.5% had CACS 1 to 99 AU, 12.1% had CACS 100 to 399 AU and 11.0% had CACS > 400 AU on gated CT (Fig. 5). The tube voltage used for non-gated CT was 100 kV for 4 studies, 120 kV for 15 studies and 80 kV/140 kV for 1 study. Two studies varied tube voltage based on body mass index and 1 study did not report the tube voltage.

Of the 3021 patient with CACS on gated CT, 129 (4.3%) showed no CAC on non-gated CT. Non-gated CT downgraded the CACS severity from > 400 to 100–399 AU for 6.4% (*n* = 44/685) and upgraded CACS severity from < 400 to > 400 AU for 1.2% (*n* = 66/5416), compared to ECG-gated CT.

**Fig. 6:**
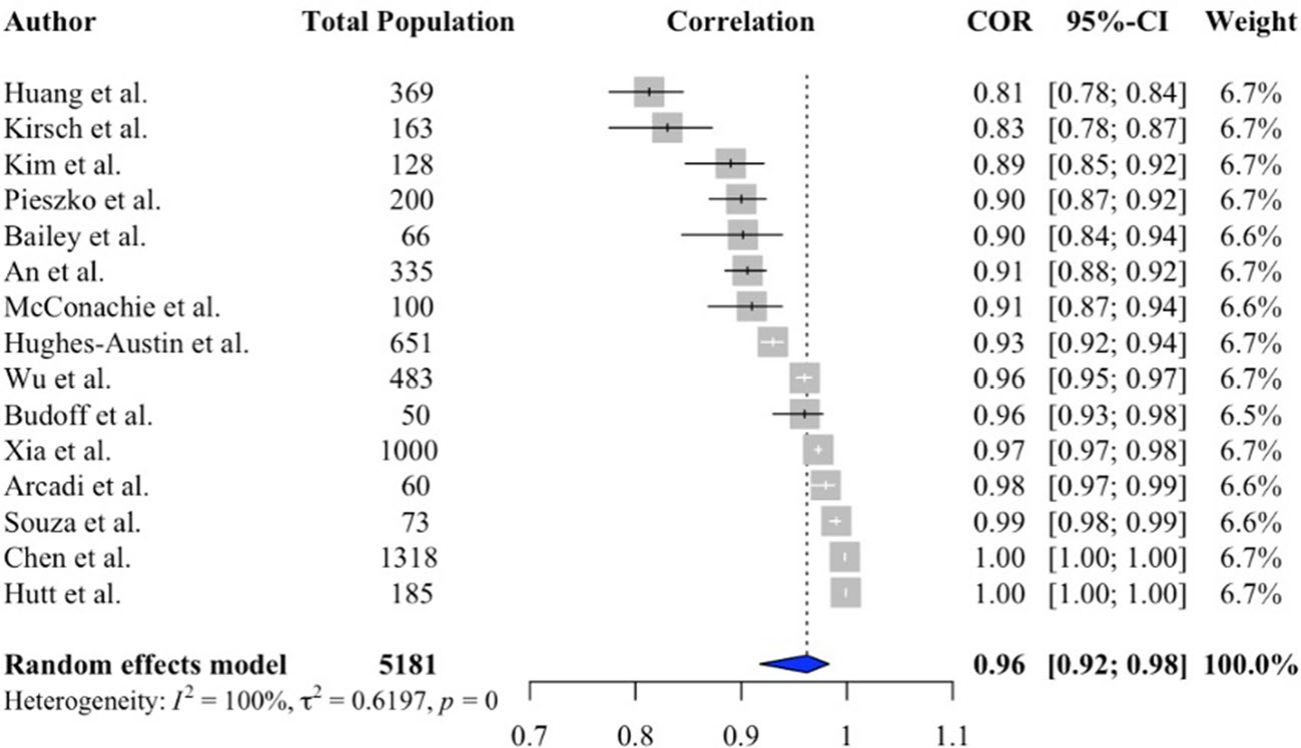
Forest plot showing the correlation between CACS assessed on ECG-gated and non-gated CT

Fifteen studies (5181 patients) provided information on the correlation between CACS calculated on non-gated and gated CT. The correlation ranged from 81 to 100% and the pooled correlation coefficient showed excellent agreement (*R* 0.96, 95% CI 0.92 to 0.98, *p* < 0.001, Fig. 6). There was significant heterogeneity between these studies (*I*^2^ > 50%, Q test *p* < 0.001) but no publication bias was identified (Eggers: *p* = 0.16; Begg: *p* = 0.30).

Seventeen studies (4598 participants) reported the comparison between gated CT and non-gated CT with CACS provided with 4 categories of severity (CAC score 0, 1–99, 100–399 and > 400 AU). The pooled Cohen κ showed very good agreement between gated and non-gated CT (0.84, 95% CI 0.75 to 0.90, *p* < 0.001, Supplementary Fig. 9). There was significant heterogeneity between studies (*I*^2^ > 50%, *Q* test *p* < 0.001) but no publication bias was identified (Eggers: *p* = 0.06; Begg: *p* = 0.93).

All studies on agreement between non-gated and gated CT were of high quality according to the QUADAS-2 scoring tool. Suboptimal scores were present in 3 domains of the QUADAS-2 tool, with 4 studies not mention whether CT interpretation was blinded and 3 studies not mention the timing of measurements (Supplementary Fig. 3).

## Discussion

In this meta-analysis, we have demonstrated that as an incidental finding on thoracic CT, coronary artery calcium is a frequent finding and has important prognostic implications. On non-gated thoracic CT scans, CAC was present on 52% of scans performed for non-cardiac indications. The occurrence of CAC on non-gated thoracic CT was related to the presence of traditional cardiovascular risk factors, except for smoking status and body mass index. Patients with incidental CAC on thoracic CT were more likely to develop subsequent cardiovascular events or all-cause mortality. Quantification of CAC on non-gated CT correlates well with the gold standard of ECG-gated CT. However, CAC on non-gated CT is only reported in approximately half of the CT scans where it is present. This highlights an important unmet need which should be addressed.

The number of thoracic CT performed around the world every year is rapidly increasing, particularly with the instigation of lung cancer screening CT. CAC will be a common finding on these CT scans, occurring in up to 91.7% of patients in some studies [52]. At present, CAC is frequently not reported on thoracic CT. In this meta-analysis, it was only reported in just over half of scans where it was present. This is a potential missed opportunity for lifestyle modification recommendations and preventative therapies. We have shown a wide range for the prevalence of CAC on non-gated thoracic CT, from 3 to 100% in studies included in this meta-analysis. Underlying differences in age, sex and prevalence of cardiovascular risk factors were key drivers of this. Patients with incidental CAC on thoracic CT were more likely to be older males, with a history of diabetes, hypercholesterolaemia and hypertension. Overall, CAC was present in just under half of patients undergoing non-gated CT. This is therefore a common incidental finding which may be a significant indicator of poor prognosis.

Although CAC is traditionally evaluated on cardiac-specific gated CT, we have confirmed that there is excellent agreement between non-gated and gated CT for the assessment of CAC. Indeed, the correlation between Agatston scores calculated using non-gated and gated CT was was excellent (Pooled *R* = 0.96) and stratification across four risk groups (CAC score 0, 1–99, 100–399 and > 400 AU) showed very good agreement (Pooled Cohen κ = 0.84). Agatston scoring is time -onsuming to perform manually on routine thoracic CT, but in the future automation with machine learning will mean that this can be done rapidly in advance of scan reporting. In the mean time, ordinal visual assessment is recommended by contemporary guidelines [10]. It should also be remembered that CAC only identifies one type of atherosclerotic plaque, and ignores non-calcified plaque.

It is interesting that we did not find an association between CAC and body mass index or smoking status. This is likely because of the complex relationship between these risk factors and the presence of cardiovascular disease and adverse cardiovascular outcomes. Large studies of unselected patients undergoing CAC scoring have shown an association between CAC and body mass index [127]. However, other studies have shown that body mass index is not an independent predictor of CAC when corrected for other cardiovascular risk factors [128], and that there is an inverse relationship between lesion-specific CAC and body mass index [129]. The lack of association between smoking status and CAC has been shown in previous studies of patients undergoing thoracic CT [130]. The reasons for this are multi-factorial, and likely include the prevalence of smoking in those undergoing imaging, the indication for imaging and challenges with recording accurate smoking status.

Absence of coronary artery calcium is associated with a low risk of cardiac events, extending to 15 years in asymptomatic patients [131]. We found that over a mean follow-up of 51.6 months, 11.9% of subjects with CAC score above 0 experienced a cardiovascular event compared to 3.4% of subjects with CAC score of 0. A previous meta-analysis demonstrated a lower risk of cardiovascular events in patients with calcium score of 0, of 0.47% over a mean follow-up of 50 months [132]. This difference is likely due to the inclusion of a wider range of patients in our meta-analysis, who had a higher frequency of underlying cardiovascular risk factors. There is also a higher risk of cardiovascular events in patietns with CAC who are undergoing lung cancer screening. These patients are more likely to be older and have a history of smoking. However, the low radiation dose nature of many lung cancer screening CT scans also means that the presence of CAC may be underestimated in these patients.

The presence of calcification on thoracic CT has important implications for patients. National and international guidelines now recommend that patients with CAC identified as an incidental finding on thoracic CT should have an assessment of their cardiovascular risk factors, such as the presence of hypertension, and be considered for preventative therapies [9, 10]. Research into the clinical implications of such management changes is currently limited. A recent single-centre retrospective analysis of 1400 chest CT found that the number needed to report to impact management was low, at only 2 scans [133]. However, to date, there are no randomised studies which assess the impact on outcomes of changing management based on the presence of incidental CAC on thoracic CT.

This study has a number of limitations. A range of different CT scanner types, radiation doses and slice thicknesses were present between studies resulting in significant heterogeneity. The number of patients who underwent both gated and non-gated CT was relatively low. A number of different calcium scoring methods were used in studies reporting prognosis and the number of cardiovascular events reported during follow-up was relatively low. The studies of prognosis reported a variety of cardiovascular events, leading to heterogeneity between these studies. All included studies were heterogenous for the participant population characteristics, imaging equipment and acquisition protocol. The random-effects model was used to compensate for some of the heterogeneity in the pooling calculation, and sensitivity analyses were used to identify influential outliers to minimise the heterogeneity.

To conclude, this meta-analysis has established that non-gated thoracic CT is a useful technique to identify incidental coronary artery calcification. CAC is frequently identified on non-gated thoracic CT and this has a significant impact on prognosis. Despite the relationship between CAC and subsequent cardiovascular events and all-cause mortality, CAC is often not mentioned in clinical reports.

### Supplementary Information

Below is the link to the electronic supplementary material.

Supplementary file1 (PDF 3076 KB)

## Data Availability

Available

## Abbreviations

ACCF/AHA: American College of Cardiology Foundation/American Heart Association
AU: Agatston units
CAC: Coronary artery calcification
CACS: Coronary artery calcium scoring
CI: Confidence intervals
CT: Computed tomography
ECG: Electrocardiogram
NIH: National Heart, Lung and Blood Institute
OR: Odds ratio
RR: Relative risk

## Declarations

### Guarantor

The scientific guarantor for this publication is Michelle C. Williams.

### Statistics and biometry

One of the authors has significant statistical expertise.

### Study subjects or cohorts overlap

When multiple publications based on the same trial were identified, only the largest sample size was included to avoid duplicate reporting.

### Methodology

- Systematic review and meta-analysis
