# Supplementary material for: Prevalence and clinical implications of coronary artery calcium scoring on non-gated thoracic computed tomography: a systematic review and meta-analysis
Source: Eur Radiol. 2023 Dec 22;34(7):4459–74. doi: 10.1007/s00330-023-10439-z (PMC11213779; doi:10.1007/s00330-023-10439-z)

Prevalence and clinical implications of coronary artery calcium scoring on non-gated thoracic computed tomography: A systematic review and meta-analysis

Electronic Supplementary Material

Eur Radiol (2023) Osborne-Grinter M, Ali A, Williams MC.

Supplementary Table 1. PRISMA Checklist

	Item #	Checklist item	Location where item is reported
**TITLE**			
Title	1	Identify the report as a systematic review.	3
**ABSTRACT**			
Abstract	2	See the PRISMA 2020 for Abstracts checklist.	39-72
**INTRODUCTION**			
Rationale	3	Describe the rationale for the review in the context of existing knowledge.	107-122
Objectives	4	Provide an explicit statement of the objective(s) or question(s) the review addresses.	124-128
**METHODS**			
Eligibility criteria	5	Specify the inclusion and exclusion criteria for the review and how studies were grouped for the syntheses.	142-153
Information sources	6	Specify all databases, registers, websites, organisations, reference lists and other sources searched or consulted to identify studies. Specify the date when each source was last searched or consulted.	134-139
Search strategy	7	Present the full search strategies for all databases, registers and websites, including any filters and limits used.	Supplementary Table 2
Selection process	8	Specify the methods used to decide whether a study met the inclusion criteria of the review, including how many reviewers screened each record and each report retrieved, whether they worked independently, and if applicable, details of automation tools used in the process.	142-153
Data collection process	9	Specify the methods used to collect data from reports, including how many reviewers collected data from each report, whether they worked independently, any processes for obtaining or confirming data from study investigators, and if applicable, details of automation tools used in the process.	156-165
Data items	10a	List and define all outcomes for which data were sought. Specify whether all results that were compatible with each outcome domain in each study were sought (e.g. for all measures, time points, analyses), and if not, the methods used to decide which results to collect.	156-172
	10b	List and define all other variables for which data were sought (e.g. participant and intervention characteristics, funding sources). Describe any assumptions made about any missing or unclear information.	156-172
Study risk of bias assessment	11	Specify the methods used to assess risk of bias in the included studies, including details of the tool(s) used, how many reviewers assessed each study and whether they worked independently, and if applicable, details of automation tools used in the process.	175-187
Effect measures	12	Specify for each outcome the effect measure(s) (e.g. risk ratio, mean difference) used in the synthesis or presentation of results.	156-172

	Item #	Checklist item	Location where item is reported
Synthesis methods	13a	Describe the processes used to decide which studies were eligible for each synthesis (e.g. tabulating the study intervention characteristics and comparing against the planned groups for each synthesis (item #5)).	190-204
	13b	Describe any methods required to prepare the data for presentation or synthesis, such as handling of missing summary statistics, or data conversions.	190-204
	13c	Describe any methods used to tabulate or visually display results of individual studies and syntheses.	190-204
	13d	Describe any methods used to synthesize results and provide a rationale for the choice(s). If meta-analysis was performed, describe the model(s), method(s) to identify the presence and extent of statistical heterogeneity, and software package(s) used.	190-204
	13e	Describe any methods used to explore possible causes of heterogeneity among study results (e.g. subgroup analysis, meta-regression).	203-204
	13f	Describe any sensitivity analyses conducted to assess robustness of the synthesized results.	203-204
Reporting bias assessment	14	Describe any methods used to assess risk of bias due to missing results in a synthesis (arising from reporting biases).	203-204
Certainty assessment	15	Describe any methods used to assess certainty (or confidence) in the body of evidence for an outcome.	190-204
**RESULTS**			
Study selection	16a	Describe the results of the search and selection process, from the number of records identified in the search to the number of studies included in the review, ideally using a flow diagram.	Figure 1
	16b	Cite studies that might appear to meet the inclusion criteria, but which were excluded, and explain why they were excluded.	Figure 1
Study characteristics	17	Cite each included study and present its characteristics.	210-225
Risk of bias in studies	18	Present assessments of risk of bias for each included study.	Supplementary Table 3
Results of individual studies	19	For all outcomes, present, for each study: (a) summary statistics for each group (where appropriate) and (b) an effect estimate and its precision (e.g. confidence/credible interval), ideally using structured tables or plots.	Supplementary Table 4, 229-231, 242-260, 264-287, 294-298, 302-321
Results of syntheses	20a	For each synthesis, briefly summarise the characteristics and risk of bias among contributing studies.	229-238, 242-247, 270-274, 279-280, 285-287, 289-290, 294-298, 314-315, 319-321, 322-325
	20b	Present results of all statistical syntheses conducted. If meta-analysis was done, present for each the summary estimate and its precision (e.g.	Figure 2,

	Item #	Checklist item	Location where item is reported
		confidence/credible interval) and measures of statistical heterogeneity. If comparing groups, describe the direction of the effect.	Figure 3, Figure 4, Figure 6, Supplementary Figure 1-9, 229-231, 249-260, 267-287, 294-298, 311-321,
	20c	Present results of all investigations of possible causes of heterogeneity among study results.	233-235, 245-247, 270-274, 279-280, 285-287, 297-298, 314-315, 319-321, 375-384
	20d	Present results of all sensitivity analyses conducted to assess the robustness of the synthesized results.	N/A
Reporting biases	21	Present assessments of risk of bias due to missing results (arising from reporting biases) for each synthesis assessed.	233-235, 245-247, 270-274, 279-280, 285-287, 297-298, 314-315, 319-321, 375-384
Certainty of evidence	22	Present assessments of certainty (or confidence) in the body of evidence for each outcome assessed.	N/A
**DISCUSSION**			
Discussion	23a	Provide a general interpretation of the results in the context of other evidence.	338-373
	23b	Discuss any limitations of the evidence included in the review.	375-384
	23c	Discuss any limitations of the review processes used.	375-384
	23d	Discuss implications of the results for practice, policy, and future research.	342-343, 388-390
**OTHER INFORMATION**			
Registration and protocol	24a	Provide registration information for the review, including register name and registration number, or state that the review was not registered.	135
	24b	Indicate where the review protocol can be accessed, or state that a protocol was not prepared.	135
	24c	Describe and explain any amendments to information provided at registration or in the protocol.	135

	Item #	Checklist item	Location where item is reported
Support	25	Describe sources of financial or non-financial support for the review, and the role of the funders or sponsors in the review.	890-891
Competing interests	26	Declare any competing interests of review authors.	893-894
Availability of data, code and other materials	27	Report which of the following are publicly available and where they can be found: template data collection forms; data extracted from included studies; data used for all analyses; analytic code; any other materials used in the review.	Supplemetary table 2, 903-904

Supplementary Table 2. Search strategy used when conducting database searches in Medline, Embase and Pubmed.

Database: Ovid MEDLINE(R) ALL <1946 to April, 2021>

Search Strategy:

("Tomography, X-Ray Computed" OR "computed tomography" OR CT OR "MDCT") AND ("untriggered" OR "ungated" OR "non-gated" OR "non-triggered" OR "non-electrocardiogram" OR “thorax" OR "chest" OR "thoracic" OR "lung" OR "pulmonary" OR "torso" OR "screening") AND ("coronary") AND ("Calcium" OR "calcification" OR "calcific" OR “calcified").

Supplementary Table 3

(A) QUADAS-2 tool to assess risk of bias in diagnostic accuracy studies

(B) NIH Quality Assessment Tool for Observational Studies

(C) American College of Cardiology Foundation/American Heart Association (ACCF/AHA) guideline for prognostic studies

(D) NIH Quality Assessment of Case-Control Studies

(A)

Author	Risk of Bias				Applicability concerns			Total
	Patient Selection	Index Test	Reference Standard	Flow and Timing	Patient Selection	Index Test	Reference Standard	
An et al. 2022	Y	Y	Y	N	Y	Y	Y	6
Arcadi et al. 2014	Y	Y	Y	Y	Y	Y	Y	7
Bailey et al. 2017	Y	Y	Y	Y	Y	Y	Y	7
Budoff et al. 2011	Y	N	N	Y	Y	Y	Y	5
Chandra et al. 2017	Y	Y	Y	Y	Y	Y	Y	7
Chen et al. 2019	Y	Y	Y	Y	Y	Y	Y	7
Chi et al. 2021	Y	N	N	N	Y	Y	Y	4
Einstein et al. 2010	Y	Y	Y	Y	Y	Y	Y	7
Fan et al. 2018	Y	Y	Y	N	Y	Y	Y	7
Fresno et al. 2022	Y	Y	Y	Y	Y	Y	Y	7
Huang et al. 2013	Y	Y	Y	Y	Y	Y	Y	7
Hutt et al. 2016	Y	Y	Y	Y	Y	Y	Y	7
Kim et al. 2008	Y	N	N	Y	Y	Y	Y	7
Kim et al. 2014	Y	Y	Y	Y	Y	Y	Y	7
Lee et al 2021.	Y	Y	Y	Y	Y	Y	Y	7
Lee SY et al 2021.	Y	N	N	Y	Y	Y	Y	5
Liu et al.2022	Y	Y	Y	Y	Y	Y	Y	7

McConachie et al. 2021	Y	Y	Y	Y	Y	Y	Y	7
Pieszko et al. 2022	Y	Y	Y	Y	Y	Y	Y	7
Souza et al. 2020	Y	Y	Y	Y	Y	Y	Y	7
Van Assen et al. 2021	Y	Y	Y	Y	Y	Y	Y	7
Wu et al. 2008	Y	Y	Y	Y	Y	Y	Y	7
Xia et al. 2019	Y	Y	Y	Y	Y	Y	Y	7
**Table 1A:** Y, Fulfilled; N, Unmet or Unclear.								

(B)

**Authors**	**1**	**2**	**3**	**4**	**5**	**6**	**7**	**8**	**9**	**10**	**11**	**12**	**13**	**14**	**Total score %**
Atkins et al. 2022	Y	Y	Y	Y	N	Y	Y	N	Y	NA	Y	N	Y	Y	77%
Aybay et al. 2021	Y	Y	Y	Y	N	Y	N	Y	Y	N	Y	N	Y	?	64%
Azour et al. 2016	Y	Y	Y	Y	N	?	Y	Y	Y	N	Y	Y	Y	N	71%
Balakrishnan et al. 2017	Y	Y	Y	Y	N	Y	Y	Y	Y	N	Y	Y	Y	N	79%
Callaway et al. 1997	Y	Y	Y	?	N	N	Y	Y	Y	N	Y	?	Y	N	64%
Chamberlin et al. 2021	Y	Y	Y	Y	N	N	?	N	Y	N	Y	N	Y	Y	57%
Chandra et al. 2021	Y	Y	Y	N	N	Y	Y	N	Y	N	Y	N	Y	Y	64%
Chen et al. 2021	Y	Y	Y	Y	N	?	N	Y	Y	N	Y	Y	Y	Y	71%
Chi et al. 2021	Y	Y	?	Y	N	N	?	N	N	N	Y	?	?	Y	36%
Choy et al 2013	Y	Y	Y	Y	N	N	N	N	N	N	Y	NA	NA	N	58%
De Mattos et al. 2022	Y	Y	Y	Y	N	Y	Y	Y	Y	N	Y	Y	Y	Y	86%
Dirrichs et al. 2015	Y	Y	Y	Y	N	Y	Y	Y	Y	N	Y	Y	Y	N	79%
Douthit et al. 2021	Y	Y	Y	Y	N	?	?	N	N	N	Y	?	Y	?	43%
Ezponda et at. 2021	Y	Y	Y	Y	N	Y	Y	Y	Y	Y	Y	?	Y	Y	86%
Foley et al. 2010	Y	Y	Y	Y	N	?	Y	Y	Y	N	Y	?	Y	N	71%
Fresno et al. 2022	Y	Y	Y	Y	N	NA	NA	NA	NA	Y	Y	Y	Y	NA	89%
Gazourian et al. 2021	Y	Y	Y	Y	N	Y	Y	Y	Y	N	Y	?	Y	Y	79%
Gernaat et al. 2016	Y	Y	Y	Y	N	?	Y	Y	Y	N	Y	Y	Y	N	71%
Gernaat et al. 2018	Y	Y	Y	Y	N	Y	Y	Y	Y	N	Y	?	Y	N	71%
Gupta et al 2018	Y	Y	Y	Y	N	Y	Y	Y	N	N	Y	?	Y	Y	71%
Hiltunen et al. 2008	Y	Y	Y	Y	N	Y	Y	Y	Y	N	Y	Y	Y	Y	86%
Htwe et al. 2015	Y	Y	Y	Y	N	Y	Y	?	Y	N	Y	Y	Y	Y	79%
Itani et al. 2003	Y	Y	Y	?	N	?	Y	Y	Y	N	Y	?	Y	N	57%

Johnson et al. 2013	Y	Y	Y	?	N	N	Y	Y	Y	N	Y	?	Y	Y	64%
Khan et al 1994	Y	Y	Y	Y	N	N	N	N	N	N	Y	?	NA	N	54%
Kirsch et al. 2012	Y	Y	Y	Y	N	Y	Y	Y	Y	N	Y	Y	Y	Y	86%
Krishnam et al 2020	Y	Y	Y	Y	N	Y	Y	Y	Y	N	Y	?	Y	Y	79%
Kucharczyk et al. 2011	Y	Y	Y	Y	N	Y	Y	Y	Y	N	Y	?	Y	N	71%
Lai et al. 2021	Y	Y	N	Y	N	Y	Y	N	Y	NA	N	N	Y	Y	62%
Lessmann et al. 2010	Y	Y	Y	Y	N	?	Y	Y	Y	N	Y	?	Y	Y	64%
Lichtenstein et al 2018	Y	Y	Y	Y	N	Y	Y	Y	Y	N	Y	?	NA	Y	85%
Machino et al. 2022	Y	Y	Y	Y	N	N	?	N	Y	N	Y	N	Y	Y	57%
MacRedmond et al. 2004	Y	Y	Y	Y	N	?	Y	Y	Y	N	Y	?	Y	N	64%
Majeed et al. 2022	Y	Y	Y	Y	N	Y	Y	Y	Y	?	Y	N	Y	Y	79%
Mascalchi et al. 2021	Y	Y	Y	Y	N	Y	Y	Y	Y	N	Y	?	Y	Y	79%
McConachie et al. 2021	Y	Y	Y	Y	N	Y	?	Y	Y	N	Y	Y	Y	Y	86%
Mendoza et al 2020	Y	Y	Y	Y	N	Y	Y	Y	Y	N	Y	N	NA	Y	77%
Muller et al. 2021	Y	Y	Y	Y	N	Y	?	Y	Y	N	Y	Y	Y	Y	79%
Nguyen et al. 2007	Y	Y	Y	Y	N	Y	Y	Y	Y	N	Y	?	Y	N	71%
Niedermeier et al. 2022	Y	Y	Y	Y	N	N	?	N	N	N	Y	N	Y	Y	50%
Ota et al.2021	Y	Y	Y	Y	N	Y	Y	Y	Y	N	Y	N	N	Y	71%
Philips et al. 2019	Y	Y	Y	Y	N	?	Y	Y	Y	N	Y	Y	Y	Y	79%
Priola et al. 2013	Y	Y	Y	Y	N	?	Y	Y	Y	N	Y	Y	Y	N	71%
Ramchand et al. 2021	Y	Y	Y	Y	N	Y	Y	Y	Y	N	Y	N	Y	Y	79%
Roehl et al. 2021	Y	Y	Y	Y	N	Y	Y	Y	Y	N	Y	N	Y	Y	79%
Schiffer et al. 2021	Y	Y	Y	Y	N	Y	Y	?	Y	N	Y	N	Y	Y	71%
Secchi et al. 2017	Y	Y	Y	Y	N	?	Y	Y	Y	N	Y	Y	Y	N	71%
Selvaraj et al. 2021	Y	Y	Y	Y	N	Y	Y	Y	Y	N	Y	Y	Y	Y	86%
Shao et al. 2014	Y	Y	N	Y	N	Y	Y	Y	Y	N	Y	Y	Y	Y	79%
Shemesh et al .2010	Y	Y	Y	?	N	?	Y	Y	Y	N	Y	?	Y	Y	64%
Sverzellati et al. 2011	Y	Y	Y	Y	N	Y	Y	Y	Y	N	Y	Y	Y	Y	86%
Trpkov et al. 2021	Y	Y	Y	Y	N	Y	Y	Y	Y	N	Y	N	Y	Y	86%
Uretsky et al. 2015	Y	Y	Y	Y	N	Y	Y	Y	Y	N	Y	Y	Y	N	79%
Vakil et al.	Y	Y	Y	Y	N	Y	?	Y	Y	N	Y	?	Y	Y	71%
Van Assen et al.	Y	Y	Y	Y	N	?	?	N	N	N	Y	?	Y	N	43%
Wang Kyle et al. 2022	Y	Y	Y	Y	N	Y	Y	Y	Y	N	Y	N	Y	Y	79%
Wang Xi et al. 2022	Y	Y	Y	Y	N	Y	Y	Y	Y	N	Y	Y	Y	Y	86%

Wenning et al. 2021	Y	Y	Y	Y	N	Y	Y	Y	Y	Y	Y	?	Y	Y	93%
West et al. 2019	Y	Y	Y	Y	Y	?	Y	Y	Y	N	Y	?	Y	N	71%
Williams et al. 2013	Y	Y	Y	Y	N	Y	Y	Y	Y	N	Y	Y	Y	N	79%
Williams et al. 2020	Y	Y	Y	Y	N	?	Y	Y	Y	N	Y	?	Y	Y	71%
Xia et al. 2019	Y	Y	Y	Y	Y	?	Y	Y	Y	N	Y	?	Y	N	71%
Yang et al. 2022	Y	Y	Y	Y	N	Y	Y	Y	Y	N	Y	N	Y	Y	79%
Yu et al. 2021	Y	Y	Y	Y	N	Y	Y	Y	Y	N	Y	N	Y	Y	79%
Zorzi et al. 2021	Y	Y	Y	Y	N	Y	Y	Y	Y	N	Y	N	Y	Y	79%

TABLE 1B: (1) Research question stated (2) Study population defined (3) Participation rate > 50% (4) Subjects selected from the same or similar population with inclusion and exclusion criteria (5) Sample size justification or power description, or variance and effect estimates (6) Exposure(s) of interest measured before outcome measurement? (7) Sufficient timeframe (8): Exposure measured as categories or continuously in relation to the outcome? (9) Independent variables clearly defined and measured in all participants (10) Exposure measured more than once (11) Outcome measures clearly defined (12) Outcome assessors blinded (13) Loss to follow up 20% or less (14) Adjusted statistics for confounding variables; Y, yes; N, no; ?, unclear; NA, not assessable

(C)

Author	**Retrospective vs Prospective Study**	**Potential for referral bias**	**Reporting coronary calcification by CHD death or myocardial infarction**	**Reporting of results by gender or ethnicity**	**Sample size greater than 1000**	**Potential for limited challenge**	**Risk factor reporting**	**Covariate or risk adjusted outcomes**	**Quality**
Aybay et al. 2021	1	1	1	1	0	2	3	0	Moderate
Barda et al. 2022	1	2	2	1	1	2	3	1	High
Bhatt et al. 2018	2	2	2	2	1	1	3	0	High
Castagna et al.2022	1	1	1	3	0	2	3	1	High
Chamberlin et al. 2021	1	1	2	1	0	1	1	1	Moderate
Chen et al.2021	1	2	2	2	0	2	3	1	High
Ezponda et at. 2021	1	2	1	1	0	2	3	1	High
Fresno et al. 2022	1	1	2	1	0	2	1	1	Moderate
Gazourian et al. 2021	1	2	2	3	1	2	3	1	High

Hasimoto et al. 2021	2	0	2	1	0	2	3	1	High
Heidinger et al. 2021	1	2	1	1	0	2	1	1	Moderate
Itani et al. 2019	2	2	2	1	1	1	3	0	High
Johnson et al. 2013	1	2	2	0	0	1	3	1	Moderate
Majeed et al. 2022	1	0	2	3	0	1	1	1	Moderate
Mascalchi et al. 2021	1	1	2	1	1	2	1	1	Moderate
O'Hare et al. 2014	1	2	2	1	0	2	3	1	High
Ota et al. 2021	1	1	1	1	0	2	3	1	Moderate
Philips et al. 2019	1	2	2	1	0	2	3	1	High
Rasmussen et al. 2015	1	2	2	1	1	1	3	1	High
Rodriquez-Granillo et al. 2017	1	2	2	0	1	1	3	1	High
Roehl et al. 2021	1	1	2	1	0	2	1	1	Moderate
Roth et al. 1997	1	2	2	1	1	1	3	0	High
Schiffer et al. 2021	1	1	2	3	0	2	1	1	High
Selvaraj et al. 2021	1	1	2	3	0	2	3	1	High
Shao et al. 2017	1	2	2	1	0	1	3	1	High
Shemesh et al. 2010	2	2	2	1	1	1	3	1	High
Sverzellati et al. 2012	2	2	2	0	1	1	3	1	High
Tahir et al. 2022	1	1	1	1	0	2	3	1	Moderate
Takei et al. 2021	1	1	1	1	0	1	1	1	Moderate
Takx et al. 2014	2	2	2	0	1	1	3	1	High
Trpkov et al. 2021	1	1	2	1	1	2	3	1	High
Wenning et al. 2021	2	1	2	1	0	2	3	1	High
Williams et al. 2012	1	2	2	1	0	1	3	1	High
Williams et al. 2019	1	2	2	0	0	1	3	1	Moderate

Williams et al. 2020	1	2	2	0	0	1	3	1	Moderate
Yang et al. 2022	1	1	2	1	1	2	3	1	High
Yu et al. 2021	1	1	2	1	0	2	1	1	Moderate
Zimmermann et al. 2020	1	2	1	1	0	2	1	1	Moderate
Zorzi et al. 2021	1	1	2	1	0	2	3	1	High

(D)

	Item1: Research question stated	Item 2: Study population defined	Item 3: Sample size justification	Item 4: Control and cases from same population?	Item 5: Reliable, valid and consistent implementation of definition, inclusion and exclusion criteria for cases and controls	Item 6: Cases clearly defined and differentiated from controls?	Item 7: If < 100% of cases/ controls were selected, were they randomly selected from those eligible?	Item 8: Use of concurrent controls?	Item 9: Confirmation that exposure/ risk occurred prior to the development of the condition/ event that defined a case	Item 10: Measures of exposure/ risk clearly defined, valid and implemented consistently across all study particpants?	Item 11: Were asssessors of exposure/ risk blinded to the case or control status of participants?	Item 12: Identification of confounding variables measured and adjusted for statistically?
Blair et al. 2014	Y	Y	?	Y	Y	Y	Y	Y	Y	Y	Y	Y
Hughes-Austin et al. 2016	Y	Y	?	Y	Y	Y	Y	Y	Y	Y	Y	Y
Van der Bijl 2016	Y	Y	?	Y	Y	Y	Y	Y	Y	Y	Y	Y
Lessman et al. 2019	Y	Y	?	Y	Y	Y	Y	Y	Y	Y	N	Y
Jacobs et al. 2011	Y	Y	Y	Y	Y	Y	Y	Y	Y	Y	Y	Y

Table 1D: Y, Yes; N, No; ?, Unclear.

Supplementary Table 4. Titles, Authors and Years of Studies Included in the Systematic Review and Meta-Analysis

Author	Year	Title
An et al.	2022	Evaluating coronary artery calcification with low-dose chest CT reconstructed by different kernels
Arcadi et al.	2014	Coronary artery calcium score on low-dose computed tomography for lung cancer screening
Atkins et al.	2022	Elevated Coronary Artery Calcium Quantified by a Validated Deep Learning Model From Lung Cancer Radiotherapy Planning Scans Predicts Mortality
Aybay et al.	2021	Ordinal Scoring of Coronary Artery Calcification byComputed Tomography Pulmonary Angiography inAcute Pulmonary Embolism
Azour et al.	2017	Estimation of cardiovascular risk on routine chest CT: Ordinal coronary artery calcium scoring as an accurate predictor of Agatston score ranges
Bailey et al.	2017	Relative predictive value of lung cancer screening CT versus myocardial perfusion attenuation correction CT in the evaluation of coronary calcium
Barda et al.	2022	Improving Cardiovascular Disease Prediction Using Automated Coronary Artery Calcium Scoring from Existing Chest CTs
Blair et al.	2014	Comparison of ordinal versus Agatston coronary calcification scoring for cardiovascular disease mortality in community-living individuals
Blakrishnan et al.	2017	Coronary artery calcification is common on nongated chest computed tomography imaging
Budoff et al.	2011	Coronary artery and thoracic calcium on noncontrast thoracic CT scans: Comparison of ungated and gated examinations in patients from the COPD Gene cohort
Calloway et al.	1997	The incidence of coronary artery calcification on standard thoracic CT scans
Castagna et al.	2022	Visual Coronary and Aortic Calcium Scoring on Chest Computed Tomography Predict Mortality in Patients With Low-Density Lipoprotein-Cholesterol ≥190 mg/dL
Chamberlin et al.	2021	Automated detection of lung nodules and coronary artery calcium using artificial intelligence on low dose CT scans for lung cancer screening: accuracy and prognostic screen
Chandra et al.	2017	Assessment of coronary artery calcium by chest CT compared with EKG-gated cardiac CT in the multicenter AIDS cohort study
Chandra et al.	2021	The Association Between Lung Hyperinflation and Coronary ArteryDisease in Smokers

Chen et al.	2021	Prognostic Value of Coronary Artery Calcification Identified by the Semi-quantitative Weston Method in the Emergency Room or Other Hospitalized Patients
Chen et al.	2019	Comparison of Nongated Chest CT and Dedicated Calcium Scoring CT for Coronary Calcium Quantification Using a 256-Dector Row CT Scanner
Chi et al.	2021	Coronary CT Calcium Score in Patients With Prior Nongated CT, Is it Necessary?
Choy et al.	2013	Pertinent reportable incidental cardiac findings on chest CT without electrocardiography gating: review of 268 consecutive cases
De Mattos et al.	2022	Computed Tomography on lung cancer screening is useful for adjuvant comorbidity diagnosis in developing countries
Dirrichs et al.	2015	Extracoronary Thoracic and Coronary Artery Calcifications on Chest CT for Lung Cancer Screening
Douthit et al.	2021	Clinical Impact of reporting Coronary Artery Calcium Scores of Non-Gated Computed Tomography on Statin Management
Einstein et al.	2010	Agreement of Visual Estimation of Coronary Artery Calcium From Low-Dose CT Attenuation Correction Scans in Hybrid PET/CT and SPECT/CT With Standard Agatston Score
Ezponda et al.	2021	Chest CT-assessed comorbidities and all-cause mortality risk in COPD patients in the BODE cohort
Fan et al.	2018	Optimized categorization algorithm of coronary artery calcification score on non-gated chest low-dose CT screening using iterative model reconstruction technique
Foley et al.	2010	Incidental cardiac findings on computed tomography imaging of the thorax
Fresno et al.	2022	Visual Ordinal Scoring of Coronary Artery Calcium on Contrast-Enhanced and Noncontrast Chest CT: A Retrospective Study of Diagnostic Performance and Prognostic Utility
Gazourian et al.	2021	Qualitative coronary artery calcificaiton scores and risk of all cause, COPD and pneumonia hospital admission in a large CT lung cancer screening cohort
Gernaat et al.	2018	Automatic quantification of calcifications in the coronary arteries and thoracic aorta on radiotherapy planning CT scans of Western and Asian breast cancer patients
Gernaat et al.	2016	Automatic Coronary Artery Calcium Scoring on Radiotherapy Planning CT Scans of Breast Cancer Patients: Reproducibility and Association with Traditional Cardiovascular Risk Factors
Gupta et al.	2018	Coronary artery calcification predicts cardiovascular complications after sepsis
Hashimoto et al.	2021	Prognostic Values of Coronary Artery Calcium and 123I-BMIPP SPECT in Patients with non-ischaemic heart failure with preserved ejection fraction

Heidinger et al.	2021	Risk assessment of acute pulmonary embolism utilizing coronary artery calcifications in patients that have undergone CT pulmonary angiography and transthoracic echocardiography
Hiltunen et al.	2008	Visual Scoring of Atherosclerosis in Chest Computed Tomography: Findings among Male Construction Workers
Htwe et al.	2015	Coronary artery calcification on low-dose computed tomography: comparison of Agatston and Ordinal Scores
Huang et al.	2013	Reliable categorisation of visual scoring of coronary artery calcification on low-dose CT for lung cancer screening: vali- dation with the standard Agatston score.
Hughes-Austin et al.	2016	Relationship of Coronary Calcium on Standard Chest CT Scans With Mortality
Hutt et al.	2016	Coronary calcium screening with dual-source CT: reliability of ungated, high-pitch chest CT in comparison with dedicated calcium-scoring CT
Itani et al.	2004	Coronary artery calcification detected by a mobile helical computed tomography unit and future cardiovascular death: 4-year follow-up of 6120 asymptomatic Japanese.
Jacobs et al.	2011	Unrequested information from routine diagnostic chest CT predicts future cardiovascular events
Johsnon et al.	2014	Coronary Artery Calcification Is Often Not Reported in Pulmonary CT Angiography in Patients With Suspected Pulmonary Embolism: An Opportunity to Improve Diagnosis of Acute Coronary Syndrome
Khan et al.	1994	Computed tomography of normal and calcified coronary arteries
Kim et al.	2014	Reliability analysis of visual ranking of coronary artery calcification on low-dose CT of the thorax for lung cancer screening: comparison with ECG-gated calcium scoring CT
Kim et al.	2008	Coronary Calcium Screening Using Low-Dose Lung Cancer Screening: Effectiveness of MDCT with Retrospective Reconstruction
Kirsch et al.	2012	Detection of coronary calcium during standard chest computed tomography correlates with multi-detector computed tomography coronary artery calcium score
Krishnam et al.	2020	Utility of routine non-gated CT chest in detection of subclinical atherosclerotic calcifications of coronary arteries in hospitalised HIV patients
Kucharczyk et al.	2011	Assessing the Impact of Incidental Findings in a Lung Cancer Screening Study by Using Low-dose Computed Tomography
Lai et al.	2021	Accelerated coronary calcium burden in breast cancer patients after radiotherapy: a comparison with age and race matched healthy women
Lee et al.	2021	Feasibility of Coronary ARtery Calcium Scoring on Dual Energy Chest Computed Tomography: A Prospective Comparison with Electrocardiogram-Gated Calcium Score Computed Tomography

Lee et al.	2021	Comparison of artery-based methods for ordinal grading of coronary artery calcium on low-dose chest computed tomography
Lessmann et al.	2019	Sex Differences in Coronary Artery and Thoracic Aorta Calcification and Their Association With Cardiovascular Mortality in Heavy Smokers
Lichtenstein et al.	2018	Correlation between coronary artery calcification by non-cardiac CT and Framingham score in young patients
Liu et al.	2022	Accuracy of non gated low dose non contract chest CT with tin filtration for coronary artery calcium scoring
Machino et al.	2022	Prevalence of coronary calcification on preoperative computed tomography and its management in thoracic surgery
MacRedmond et al.	2004	Screening for lung cancer using low dose CT scanning.
Majeed et al.	2022	Frequency of Statin Prescription Among Individuals with Coronary Calcifications Detected Through LungCancer Screening
Mascalchi et al.	2021	Moderate-Severe Coronary Calcification predicts long-term cardiovascular death in CT lung cancer screening: the ITALUNG trial
McConachie et al.	2021	Accurate measurement of coronary artery calcium in cancer patients using the CT component of PET/CT scans
Mendoza et al.	2020	Impact of Significant Coronary Artery Calcification Reported on Low-Dose Computed Tomography Lung Cancer Screening
Mets et al.	2013	Lung Cancer Screening CT-Based Prediction of Cardiovascular Events
Muller et al.	2021	Prevalence of incidental premature calcification in an HIV-infected South African population using conventional computed tomography chest radiography
Neidermeier et al.	2022	Incidental CT findings in the Elderly with Low Energy Falls: Prevalence and Implications
Nguyen et al.	2007	Prevalence and determinants of coronary and aortic calcifications assessed by chest CT in renal transplant recipients.
O'Hare et al.	2014	Coronary Artery Calcification on Computed Tomography Correlates With Mortality in Chronic Obstructive Pulmonary Disease
Ohmoto-Sekine et al.	2016	Prevalence and distribution of coronary calcium in asymptomatic Japanese subjects in lung cancer screening computed tomography
Ota et al.	2021	Association between coronary artery calcium score on non-contrast chest computed tomography and all cause mortality among patients with congestive heart failure

Phillips et al.	2019	Comparison of Framingham risk score and chest-CT identified coronary artery calcification in breast cancer patients to predict cardiovascular events
Phillips et al.	2018	Reporting of coronary artery calcification on chest CT studies in breast cancer patients at high risk of cancer therapy related cardiac events
Pieszko et al.	2022	Reproducibility of quantitative coronary calcium scoring from PET/CT attenuation maps: comparison to ECG-gated CT scans
Priola et al.	2013	Clinical Implications and Added Costs of Incidental Findings in an Early Detection Study of Lung Cancer by Using Low-Dose Spiral Computed Tomography
Ramchand et al.	2021	Incidental Thoracic Aortic Dilation on Chest Computed Tomography in Patients with Atrial Fibrillation
Rasmussen et al. .	2015	Coronary artery calcification detected in lung cancer screening predicts cardiovascular death
Reiter et al.	2018	Frequency and distribution of incidental findings deemed appropriate for S modifier designation on low-dose CT in a lung cancer screening program
Rodriguez-Granillo et al.	2017	Impact on mortality of coronary and non-coronary cardiovascular findings in non-gated thoracic CT by malignancy status
Roehl et al.	2021	Cardiovascular Evaluation of Liver Transplant Patients by using Coronary Calcium Scoring in ECG-Synchronised Computed Scans
Roth et al.	1997	Coronary artery calcification at CT as a predictor for cardiac complications of thoracic surgery.
Schiffer et al.	2021	Coronary and aortic calcification are associated with cardiovascular events on immune checkpoint inhibitor therapy
Secchi et al.	2017	Detection of incidental cardiac findings in noncardiac chest computed tomography
Selvaraj et al.	2021	Incremental prognostic value of visually estimated coronary artery calcium in patients undergoing positron emission tomography imaging
Shao et al.	2017	Prognostic value of visually detected coronary artery calcification on unenhanced non-gated thoracic computed tomography for prediction of non-fatal myocardial infarction and all-cause mortality
Shemesh et al.	2006	Frequency of coronary artery calcification on low-dose computed tomography screening for lung cancer
Shemesh et al.	2010	Ordinal Scoring of Coronary Artery Calcifications on Low-Dose CT Scans of the Chest is Predictive of Death from Cardiovascular Disease
Shipe et al.	2021	Preoperative coronary artery calcifications in veterans predict higher all cause mortality in early stage lung cancer: a cohort study

Souza et al.	2020	Quantification of Calcified Coronary Plaques by Chest Computed Tomography: Correlation with the Calcium Score Technique
Suh et al.	2020	Coronary artery calcium severity grading on non-ECG-gated low-dose chest computed tomography: a multiple-observer study in a nationwide lung cancer screening registry
Sverzellati et al.	2016	Under-reporting of cardiovascular findings on chest CT
Sverzellati et al.	2012	Relationship and Prognostic Value of Modified Coronary Artery Calcium Score, FEV1, and Emphysema in Lung Cancer Screening Population: The MILD Trial
Tahir et al.	2022	Utility of Noncancerous Chest CT Features for Predicting Overall Survival and Noncancer Death in Patients With Stage I Lung Cancer Treated With Stereotactic Body Radiotherapy
Takei et al.	2021	Combined assessment of pulmonary arterial enlargement and coronary calcification predicts the prognosis of patients with chronic obstructive pulmonary disease
Trypkov etl al.	2021	Visually estimated coronary artery calcium score improves SPECT-MPI risk stratification
Uretsky et al.	2015	The Interplay of Physician Awareness and Reporting of Incidentally Found Coronary Artery Calcium on the Clinical Management of Patients Who Underwent Noncontrast Chest Computed Tomography
Vakil et al.	2002	Predictive Value of Coronary Artery Calcium in Patients Receiving Computed Tomography Pulmonary Angiography for Suspected Pulmonary Embolism in the Emergency Department
Van Assens et al.	2021	Automatic coronary calcium scoring in chest CT using a deep neural network in direct comparison with non contrast cardiac CT: A validation study
Van der Bijl et al.	2016	Coronary or thoracic artery calcium score in provoked and unprovoked pulmonary embolism: a case-control stud
Velangi et al.	2021	Impact of the 2016 SCCT/STR guidelines for coronary artery calcium scoring on noncardiac chest CT scans on lung cancer screening CT reporting
Wang et al.	2022	Coronary Artery Calcifications and Cardiac Risk after Radiation Therapy for Stage III Lung Cancer
Wang et al.	2022	Incremental Value of Noncontrast Chest Computed Tomography-derived Parameters in Predicting Subclinical Carotid Atherosclerosis
Wenning et al.	2021	Coronary artery calcium burden, carotid atherosclerotic plaque burden, and myocardial blood flow in patients with end-stage renal disease: A non-invasive imaging study combining PET/CT and 3D ultrasound
West et al.	2019	Significance of Coronary Artery Calcium Found on Non−Electrocardiogram-Gated Computed Tomography During Preoperative Evaluation for Liver Transplant

Williams et al.	2020	Coronary Artery Calcification on Thoracic Computed Tomography Is an Independent Predictor of Mortality in Patients With Bronchiectasis
Williams et al.	2013	Frequency of unrecognized, unreported, or underreported coronary artery and cardiovascular calcification on noncardiac chest CT
Williams et al.	2019	Coronary artery calcification is associated with mortality independent of pulmonary embolism severity: a retrospective cohort study
Williams et al.	2012	Coronary artery calcification is increased in patients with COPD and associated with increased morbidity and mortality
Wu et al.	2008	Coronary Arterial Calcification on Low-Dose Ungated MDCT for Lung Cancer Screening: Concordance Study with Dedicated Cardiac CT
Xia et al.	2019	High-pitch dual-source CT for coronary artery calcium scoring: A head-to- head comparison of non-triggered chest versus triggered cardiac acquisition
Yang et al.	2022	Nongated Computed Tomography Predicts Perioperative Cardiovascular Risk in Lung Cancer Surgery
Yu et al.	2021	Incidentally identified coronary artery calcium on non-contrast CT scan of the chest predicts major adverse cardiac events among hospital inpatents
Zimmerman et al.	2020	Coronary calcium scoring assessed on native screening chest CT imaging as predictor for outcome in COVID-19: An analysis of a hospitalized German cohort
Zorzi et al.	2021	Coronary artery calcium on standard chest computed tomography predicts cardiovascular events after liver transplantation

Supplementary Figure 1. Forest plot of the standardised mean difference in age between patients with a coronary artery calcium score (CACS) 0 and CACS >0.
(CI, confidence interval, SD, standard deviation).

Author	No. CACS>0	CACS>0 Mean Age	SD	No. CACS 0	CACS 0 Mean Age	SD	SMD	95%-CI	Weight
Trypkov etl al.	3293	68.4	10.6	1427	68.5	11.3	-0.0	[-0.1; 0.1]	7.4%
Lessmann et al.	4318	62.0	8.7	1400	58.6	6.0	0.4	[0.4; 0.5]	7.4%
Lichtenstein et al.	19	41.5	6.7	143	35.8	9.5	0.6	[0.1; 1.1]	5.3%
Chamberlin et al.	86	67.7	5.7	31	64.2	5.0	0.6	[0.2; 1.0]	5.8%
Chandra et al.	406	66.2	6.2	75	61.8	5.0	0.7	[0.5; 1.0]	6.8%
Shao et al.	185	64.3	9.4	225	56.8	9.2	0.8	[0.6; 1.0]	7.0%
Ota et al.	346	76.3	11.0	204	66.1	14.9	0.8	[0.6; 1.0]	7.1%
Chandra et al.	1590	63.3	8.6	990	56.3	8.0	0.8	[0.8; 0.9]	7.4%
Krishnam et al.	41	53.9	10.9	102	43.4	11.0	1.0	[0.6; 1.3]	6.0%
Balakrishnan et al.	204	73.6	8.8	100	64.8	8.6	1.0	[0.8; 1.3]	6.7%
Williams et al.	196	72.3	8.4	166	59.0	15.0	1.1	[0.9; 1.3]	6.9%
Kirsch et al.	49	57.0	8.0	114	48.0	8.0	1.1	[0.8; 1.5]	6.1%
Phillips et al.	66	69.2	12.0	190	54.8	12.0	1.2	[0.9; 1.5]	6.5%
Gupta et al.	167	65.0	11.4	100	48.0	13.6	1.4	[1.1; 1.7]	6.6%
Johnson et al.	206	66.6	12.6	263	43.7	14.4	1.7	[1.5; 1.9]	6.9%
**Random effects model**	**11172**			**5530**			**0.9**	**[0.7; 1.1]**	**100.0%**
**Prediction interval**								**[0.0; 1.8]**	

Heterogeneity: $I^2 = 98\%$, $\tau^2 = 0.1529$, $p < 0.01$

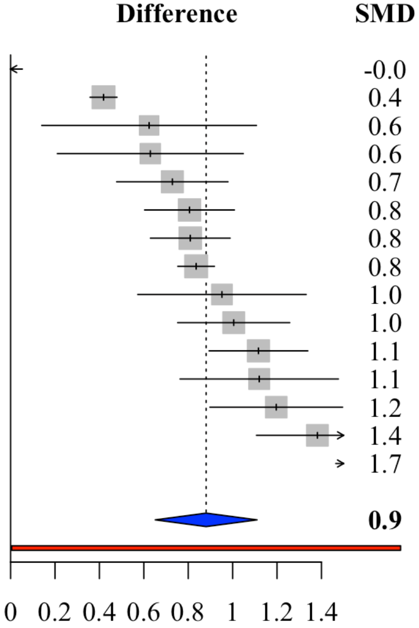

Supplementary Figure 2. Forest Plot of the odds ratio (OR) of being male in patients with coronary artery calcium score (CACS) 0 and CACS > 0 groups.
(No., Number; CI, confidence interval).

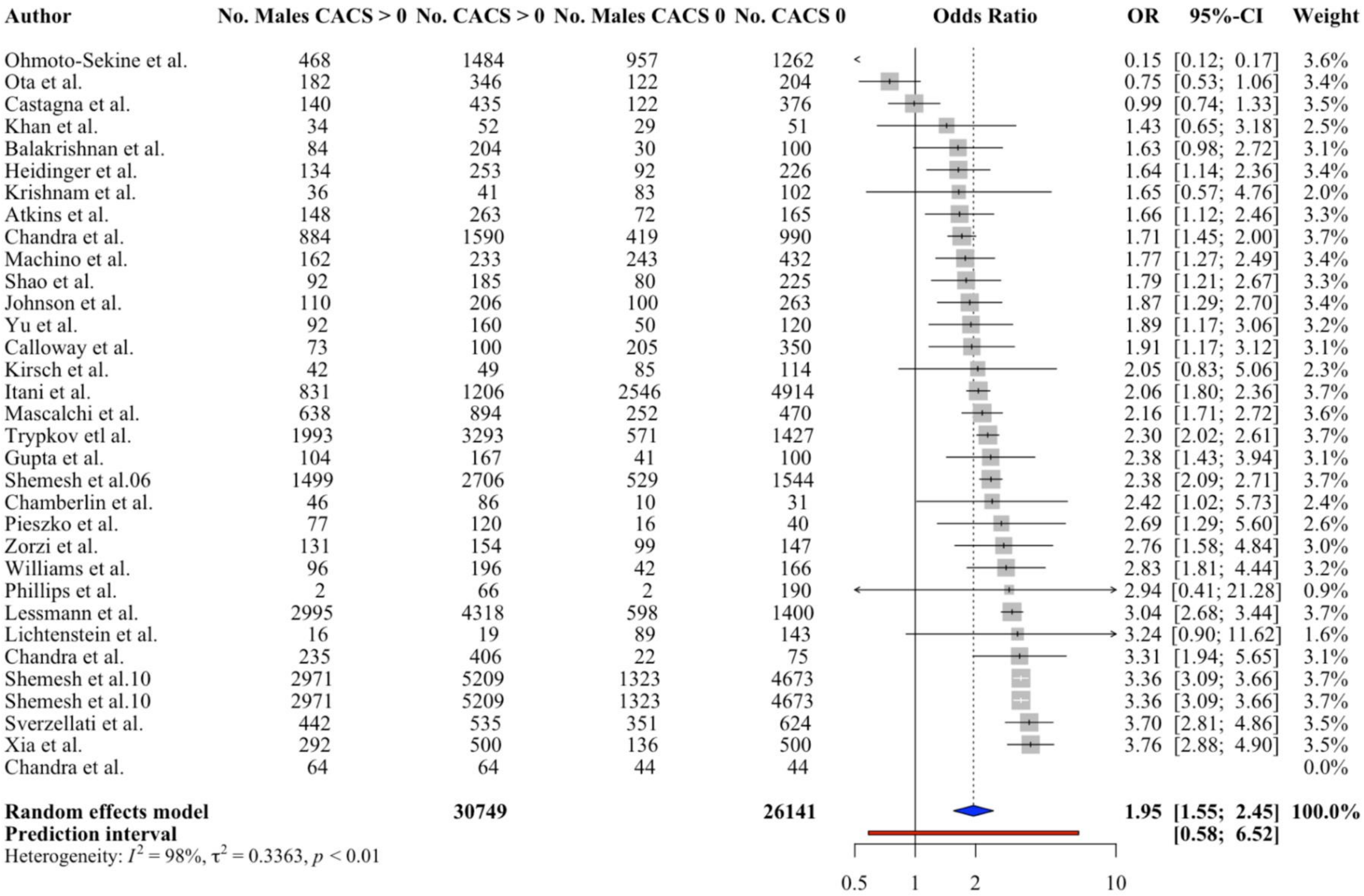

Supplementary Figure 3. Forest plot of the odds ratio (OR) of having diabetes in patients with coronary artery calcium score (CACS) 0 and CACS >0.
(CI, confidence interval)

Author	No. Diabetes CACS > 0	No. CACS > 0	No. Diabetes CACS 0	No. CACS 0	OR	95%-CI	Weight
Chandra et al.	5	64	5.0	44	0.66	[0.18; 2.43]	2.6%
Lessmann et al.	182	4318	79.0	1400	0.74	[0.56; 0.97]	5.4%
Pieszko et al.	41	120	12.0	40	1.21	[0.56; 2.63]	4.0%
Balakrishnan et al.	45	204	17.0	100	1.38	[0.74; 2.56]	4.5%
Williams et al.	21	196	11.0	166	1.69	[0.79; 3.62]	4.1%
Shemesh et al.06	34	2706	10.0	1544	1.95	[0.96; 3.96]	4.2%
Chamberlin et al.	28	86	6.0	31	2.01	[0.74; 5.46]	3.4%
Castagna et al.	113	435	53.0	376	2.14	[1.49; 3.07]	5.2%
Trypkov etl al.	969	3293	229.0	1427	2.18	[1.86; 2.56]	5.5%
Krishnam et al.	10	41	13.0	102	2.21	[0.88; 5.54]	3.6%
Ota et al.	127	346	42.0	204	2.24	[1.49; 3.35]	5.1%
Zorzi et al.	50	154	26.0	147	2.24	[1.30; 3.85]	4.7%
Chandra et al.	141	1590	41.0	990	2.25	[1.58; 3.22]	5.2%
Gupta et al.	72	167	24.0	100	2.40	[1.38; 4.17]	4.7%
Heidinger et al.	61	253	24.0	226	2.67	[1.60; 4.46]	4.8%
Mascalchi et al.	87	894	17.0	470	2.87	[1.69; 4.89]	4.7%
Atkins et al.	46	263	9.0	165	3.67	[1.75; 7.73]	4.1%
Kirsch et al.	2	49	1.0	114	4.81	[0.43; 54.32]	1.1%
Yu et al.	64	160	14.0	120	5.05	[2.66; 9.58]	4.4%
Johnson et al.	59	206	19.0	263	5.15	[2.96; 8.99]	4.7%
Phillips et al.	15	66	7.0	190	7.69	[2.98; 19.87]	3.5%
Machino et al.	56	233	16.4	432	8.02	[4.50; 14.29]	4.6%
Chandra et al.	43	406	1.0	75	8.77	[1.19; 64.66]	1.5%
Shao et al.	71	185	11.0	225	12.12	[6.17; 23.79]	4.3%
**Random effects model**		**16435**		**8951**	**2.63**	**[1.95; 3.54]**	**100.0%**
**Prediction interval**						**[0.69; 9.98]**	

Heterogeneity: $I^2 = 84\%$, $\tau^2 = 0.3930$, $p < 0.01$

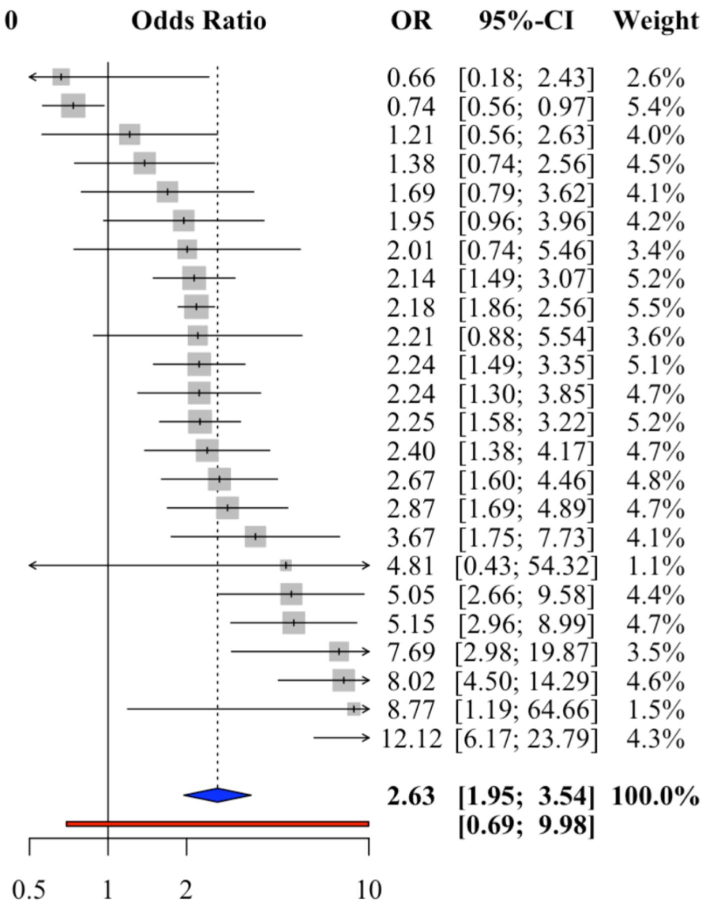

Supplementary Figure 4. Forest plot of the odds ratio (OR) of having hypercholesterolaemia in patients with coronary artery calcium score (CACS) 0 and CACS >0 groups.
(CI, confidence interval)

Author	No. Hypercholesterolaemia CACS > 0	No. CACS > 0	No. Hypercholesterolaemia CACS 0	No. CACS 0	Odds Ratio	OR	95%-CI	Weight
Zorzi et al.	10	154	44	147		0.16	[0.08; 0.34]	4.6%
Ota et al.	76	346	101	204		0.29	[0.20; 0.42]	4.9%
Machino et al.	61	233	177	432		0.51	[0.36; 0.72]	4.9%
Mascalchi et al.	196	894	108	470		0.94	[0.72; 1.23]	5.0%
Chamberlin et al.	63	86	23	31		0.95	[0.37; 2.43]	4.3%
Trypkov etl al.	1757	3293	646	1427		1.38	[1.22; 1.57]	5.0%
Chandra et al.	53	64	34	44		1.42	[0.54; 3.70]	4.3%
Chandra et al.	541	1590	224	990		1.76	[1.47; 2.11]	5.0%
Pieszko et al.	92	120	24	40		2.19	[1.02; 4.69]	4.5%
Balakrishnan et al.	124	204	41	100		2.23	[1.37; 3.63]	4.8%
Atkins et al.	148	263	56	165		2.50	[1.67; 3.75]	4.9%
Castagna et al.	357	435	241	376		2.56	[1.86; 3.54]	5.0%
Yu et al.	73	160	28	120		2.76	[1.63; 4.66]	4.8%
Gupta et al.	77	167	22	100		3.03	[1.73; 5.32]	4.8%
Heidinger et al.	133	253	50	226		3.90	[2.62; 5.82]	4.9%
Williams et al.	77	196	18	166		5.32	[3.02; 9.38]	4.8%
Kirsch et al.	34	49	32	114		5.81	[2.79; 12.08]	4.6%
Johnson et al.	105	206	34	263		7.00	[4.46; 11.00]	4.9%
Shao et al.	97	185	28	225		7.76	[4.75; 12.66]	4.8%
Chandra et al.	240	406	8	75		12.11	[5.67; 25.88]	4.5%
Phillips et al.	49	66	20	190		24.50	[11.92; 50.36]	4.6%
**Random effects model**		**9370**		**5905**		**2.28**	**[1.33; 3.93]**	**100.0%**
**Prediction interval**							**[0.19; 27.36]**	

Heterogeneity: $I^2 = 95\%$, $\tau^2 = 1.3405$, $p < 0.01$

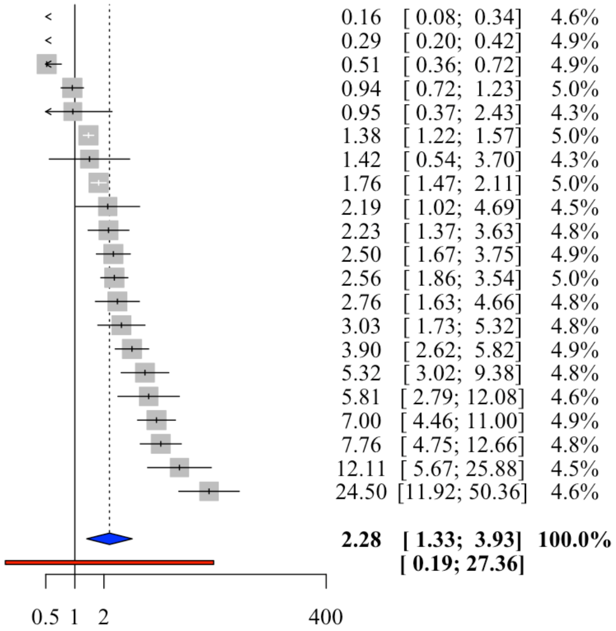

Supplementary Figure 5. Forest plot of the odds ratio (OR) of having hypertension in patients with coronary artery calcium score (CACS) 0 and CACS >0.
(CI, confidence interval)

Author	No. Hypertension CACS > 0	No. CACS > 0	No. Hypertension CACS 0	No. CACS 0	OR	95%-CI	Weight
Balakrishnan et al.	72	204	57	100	0.41	[0.25; 0.67]	4.5%
Lessmann et al.	705	4318	350	1400	0.59	[0.51; 0.68]	4.6%
Castagna et al.	343	435	305	376	0.87	[0.61; 1.23]	4.5%
Chandra et al.	188	406	35	75	0.99	[0.60; 1.61]	4.5%
Gupta et al.	84	167	41	100	1.46	[0.88; 2.40]	4.4%
Chandra et al.	35	64	19	44	1.59	[0.73; 3.44]	4.2%
Chamberlin et al.	64	86	18	31	2.10	[0.89; 4.98]	4.1%
Krishnam et al.	9	41	10	102	2.59	[0.97; 6.94]	4.0%
Chandra et al.	699	1590	221	990	2.73	[2.28; 3.27]	4.6%
Kirsch et al.	8	49	7	114	2.98	[1.02; 8.75]	3.9%
Williams et al.	54	196	18	166	3.13	[1.75; 5.59]	4.4%
Heidinger et al.	190	253	103	226	3.60	[2.45; 5.30]	4.5%
Atkins et al.	165	263	52	165	3.66	[2.42; 5.53]	4.5%
Trypkov etl al.	2098	3293	446	1427	3.86	[3.38; 4.41]	4.6%
Machino et al.	132	233	87	432	5.18	[3.65; 7.35]	4.5%
Mascalchi et al.	384	894	54	470	5.80	[4.24; 7.93]	4.6%
Shao et al.	129	185	48	225	8.49	[5.43; 13.29]	4.5%
Johnson et al.	146	206	56	263	8.99	[5.90; 13.71]	4.5%
Phillips et al.	42	66	18	190	16.72	[8.32; 33.61]	4.3%
Yu et al.	121	160	17	120	18.80	[10.04; 35.20]	4.3%
Ota et al.	247	346	23	204	19.63	[12.00; 32.13]	4.5%
Zorzi et al.	67	154	4	147	27.53	[9.70; 78.16]	3.9%
Pieszko et al.	117	120	15	40	65.00	[17.49; 241.54]	3.6%
**Random effects model**		**13729**		**7407**	**3.89**	**[2.26; 6.70]**	**100.0%**
**Prediction interval**						**[0.29; 51.75]**	

Heterogeneity: $I^2 = 97\%$, $\tau^2 = 1.4792$, $p < 0.01$

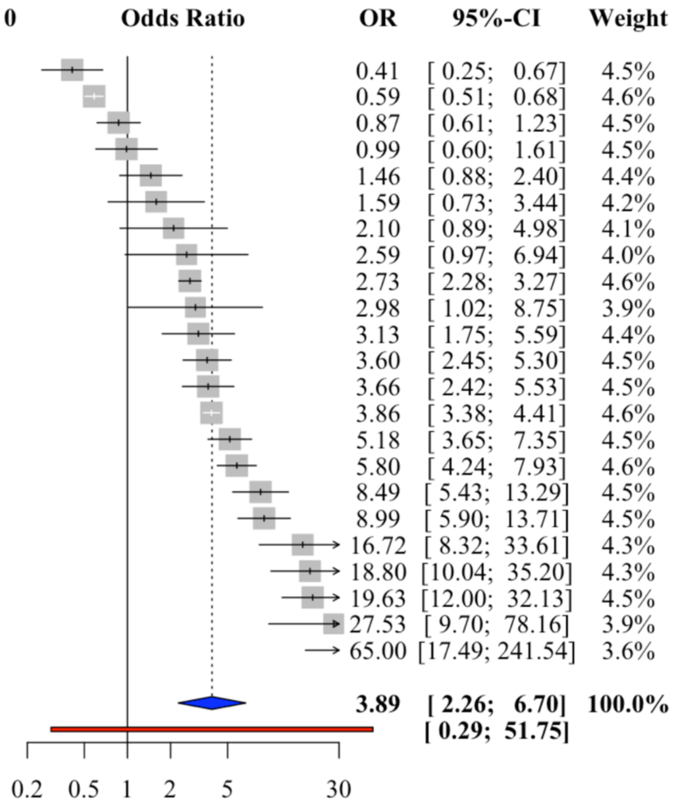

Supplementary Figure 6. Forest plot of the odds ratio (OR) of having a smoking history in patients with coronary artery calcium score (CACS) 0 and CACS >0.
(CI, confidence interval)

Author	No. Smokers CACS > 0	No. CACS > 0	No. Smokers CACS 0	No. CACS 0	OR	95%-CI	Weight
Pieszko et al.	13	120	8	40	0.49	[0.19; 1.28]	3.8%
Chandra et al.	608	1590	540	990	0.52	[0.44; 0.61]	5.8%
Chamberlin et al.	35	86	17	31	0.57	[0.25; 1.29]	4.2%
Krishnam et al.	11	41	35	102	0.70	[0.31; 1.57]	4.2%
Kirsch et al.	11	49	33	114	0.71	[0.32; 1.56]	4.3%
Chandra et al.	179	406	35	75	0.90	[0.55; 1.48]	5.1%
Lessmann et al.	2160	4318	723	1400	0.94	[0.83; 1.06]	5.8%
Ota et al.	194	346	113	204	1.03	[0.73; 1.46]	5.5%
Zorzi et al.	36	154	33	147	1.05	[0.62; 1.80]	5.0%
Trypkov etl al.	368	3293	143	1427	1.13	[0.92; 1.39]	5.7%
Chandra et al.	18	64	11	44	1.17	[0.49; 2.81]	4.0%
Balakrishnan et al.	118	204	49	100	1.43	[0.88; 2.31]	5.2%
Machino et al.	162	233	243	432	1.77	[1.27; 2.49]	5.5%
Heidinger et al.	137	253	86	226	1.92	[1.33; 2.77]	5.4%
Yu et al.	63	160	30	120	1.95	[1.16; 3.28]	5.1%
Williams et al.	108	196	63	166	2.01	[1.32; 3.06]	5.3%
Shao et al.	115	185	92	225	2.38	[1.59; 3.54]	5.4%
Atkins et al.	248	263	143	165	2.54	[1.28; 5.06]	4.6%
Lichtenstein et al.	11	19	48	143	2.72	[1.03; 7.21]	3.7%
Phillips et al.	31	66	24	190	6.13	[3.21; 11.68]	4.7%
Johnson et al.	10	206	1	263	13.37	[1.70; 105.29]	1.7%
Mascalchi et al.	894	894	470	470			0.0%
Shemesh et al.10	5209	5209	4673	4673			0.0%
**Random effects model**		**18355**		**11747**	**1.35**	**[0.98; 1.85]**	**100.0%**
**Prediction interval**						**[0.33; 5.56]**	

Heterogeneity: $I^2 = 89\%$, $\tau^2 = 0.4339$, $p < 0.01$

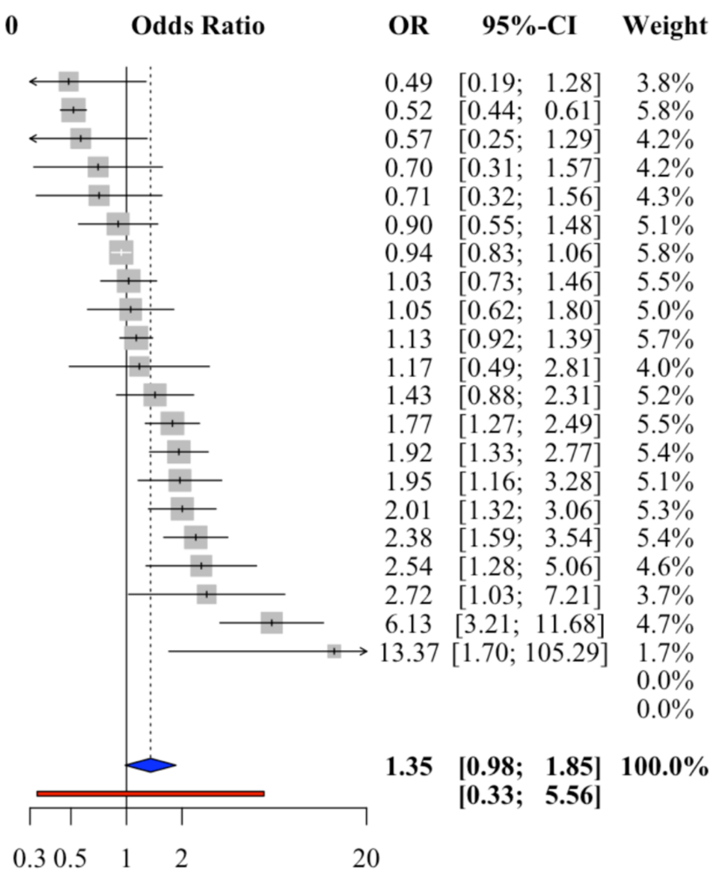

Supplementary Figure 7. Forest plot of the standardised mean difference (SMD) in body mass index (BMI) between patients with coronary artery calcium score (CACS) 0 and CACS > 0 groups.
(SD, standard deviation; CI, confidence interval)

Author	No. CACS > 0	CACS > 0 Mean BMI	CACS > 0 SD	No. CACS 0	CACS 0 Mean BMI	CACS 0 SD	SMD	95%-CI	Weight
Krishnam et al.	41	23.6	4.7	102	26.4	5.8	-0.51	[-0.87; -0.14]	16.7%
Balakrishnan et al.	204	27.1	6.7	100	27.1	5.8	0.00	[-0.24; 0.24]	20.0%
Lessmann et al.	4318	27.1	6.2	1400	26.8	6.4	0.05	[-0.01; 0.11]	23.3%
Kirsch et al.	49	28.0	4.0	114	27.0	4.0	0.25	[-0.09; 0.58]	17.5%
Xia et al.	500	26.7	4.1	500	25.5	2.5	0.35	[0.23; 0.48]	22.5%
**Random effects model**	**5112**			**2216**			**0.05**	**[-0.34; 0.44]**	**100.0%**
**Prediction interval**								**[-0.98; 1.08]**	

Heterogeneity: $I^2 = 87\%$, $\tau^2 = 0.0850$, $p < 0.01$

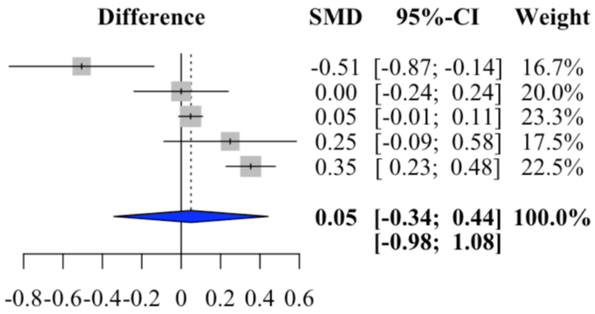

Supplementary Figure 8. Forest plot of the pooled relative risk (RR) for pooled events between high and low coronary artery calcium score (CACS) groups.

(CI, confidence interval).

Author	High CACS Events	No. High CACS	CACS 0 Events	No. CACS 0	RR	95%-CI	Weight
Schiffer et al.	17	21	14	27	1.56	[1.03; 2.37]	12.1%
Jacobs et al.	191	579	62	461	2.45	[1.89; 3.18]	13.1%
Aybay et al.	3	16	13	214	3.09	[0.98; 9.73]	6.9%
Yang et al.	7	134	53	3421	3.37	[1.56; 7.28]	9.5%
Zorzi et al.	7	26	11	147	3.60	[1.54; 8.43]	8.9%
Williams et al.	45	79	20	129	3.67	[2.35; 5.74]	12.0%
Castagna et al.	24	76	29	376	4.09	[2.53; 6.63]	11.7%
Trypkov etl al.	211	1032	44	1427	6.63	[4.84; 9.08]	12.8%
Shemesh et al.	84	625	43	3673	11.48	[8.03; 16.42]	12.5%
Roth et al.	10	46	0	27	59.37	[0.12; 29563.75]	0.5%
Shao et al.	23	28	.	225			0.0%
**Random effects model**		**2662**		**10127**	**3.92**	**[2.50; 6.14]**	**100.0%**
**Prediction interval**						**[0.94; 16.40]**	

Heterogeneity: $I^2 = 88\%$, $\tau^2 = 0.3458$, $p < 0.01$

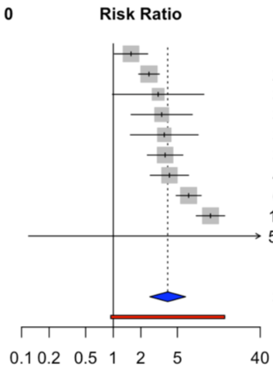

Supplementary Figure 9. Forest plot showing agreement between gated and non-gated CT in studies where coronary artery calcium score was provided with 4 categories of severity.

(COR, pooled Cohen κ; CI, confidence interval)

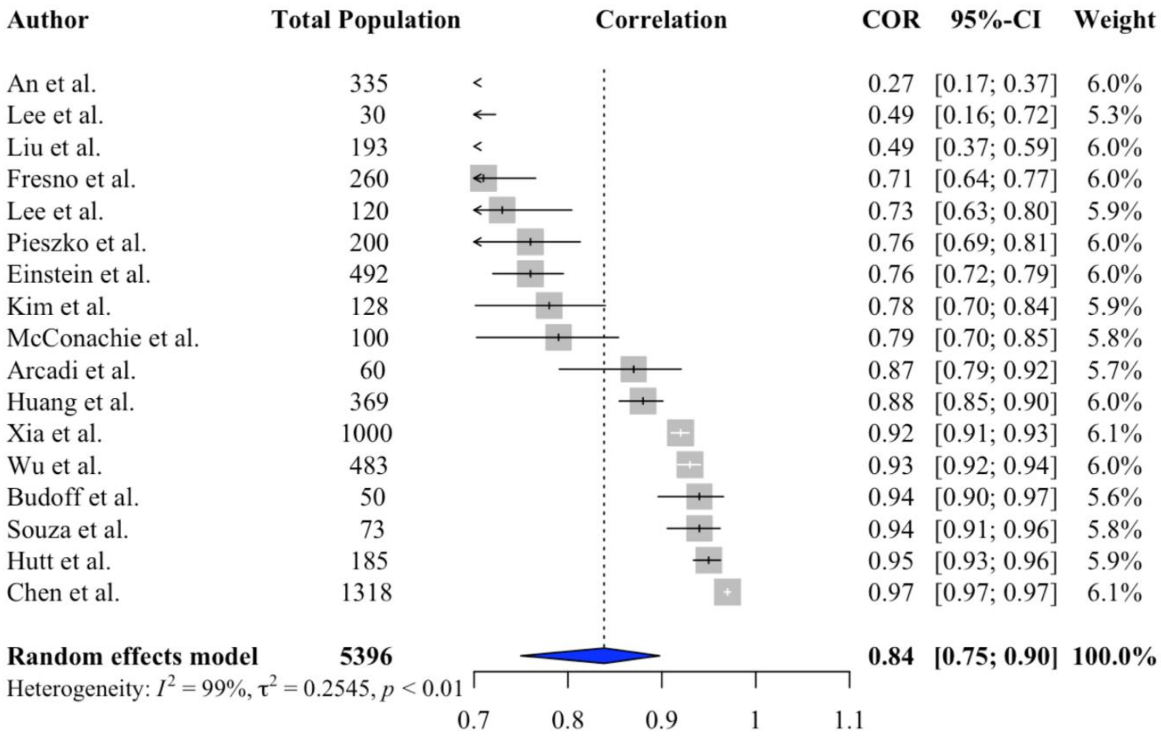

Supplement: Supplementary file 1 — Supplementary file1 (PDF 3076 KB) [file 330_2023_10439_MOESM1_ESM.pdf]
